# Relationship of Luminescent, Thermo-Oxidative and Photocatalytic Properties of ZnO Micro and Nanostructures

**DOI:** 10.3390/molecules31162793

**Published:** 2026-08-11

**Authors:** Makhach Gadzhiev, Elena Vorobyova, Valeriya Krasnova, Nadezhda Aluker, Arsen Muslimov, Sergey Antipov, Maksim Il’ichev, Yury Kulikov, Andrey Chistolinov, Damir Yusupov, Ivan Volchkov, Alexander Tyuftyaev, Vladimir Kanevsky

**Affiliations:** 1 Joint Institute for High Temperatures, Russian Academy of Sciences, 125412 Moscow, Russia; sappy78@mail.ru (S.A.);; 2 Department of Chemistry, Francisk Skorina Gomel State University, 246028 Gomel, Belarus; evorobyova@gsu.by; 3 Complex of Crystallography and Photonics, National Research Centre “Kurchatov Institute”, 119333 Moscow, Russia; 4 Federal Research Center of Coal and Coal Chemistry, Siberian Branch of the RAS, 650000 Kemerovo, Russia; naluker@gmail.com

**Keywords:** zinc oxide, luminescence, nanoparticles, microparticles, photocatalysis, thermal oxidation, solar radiation, methylene blue photodegradation, polyethylene, simulated sunlight

## Abstract

In this work, a comprehensive analysis of the relationship between photoluminescent, thermo-oxidative, and photocatalytic (upon simulated sunlight exposure) properties of ZnO powders is performed. The correlation between the X-ray diffraction and microscopic data is studied. ZnO powders of various sizes and morphologies were used: pseudo-spherical nanoparticles (30–50 nm), submicron faceted crystallites (100–500 nm), and plate- and rod-like microstructures (up to 20 μm). The mean specific surface area values were 32 m^2^/g, 3.8 m^2^/g, and 2.6 m^2^/g for pseudo-spherical nanoparticles, submicron faceted crystallites, and plate- and rod-like microstructures, respectively. According to the XRD data, microstresses and carbon-based impurities were present in ZnO nanoparticles, which is characteristic of nanomaterials synthesized at low temperatures. According to the photoluminescence spectroscopy data, the emission in ZnO was reduced due to high defectiveness, and characteristic emission bands indicated the presence of organic impurities. Upon long signal registration times, an intensive luminescence band with an effective maximum at 579 nm occurred, which indicated the presence of long-term components exhibiting decay times τ ~300 μs. According to the XRD data, the crystal structure parameters of ZnO submicro- and microparticles were close, with no impurities present. In their photoluminescence spectra, pronounced UV and defect-related bands were present with intensity ratios of 11.6 and 6.88, respectively. The decrease in the UV and defect-related luminescence band intensity ratios indicates deviation from the stoichiometry toward an increased Zn over oxygen content. At long signal registration times, in submicron ZnO particles, a luminescence band with maxima at 425 and 490 nm is present, which decays rapidly. An emission band in the 530 nm region is also present, which decays for ≤80 μs, and a weak long-wavelength emission decaying for ~100 μs. At long delay and strobe times (up to milliseconds), only an emission in the 460 nm region is observed, which we connect to the triplet–singlet transition of a defect center (F*, F^+^*). At lower intensities, an emission connected to the surface contamination by organic impurities is observed. In photoluminescence spectra of ZnO microparticles, no long-wavelength emission components are observed. However, upon immersing into methylene blue solution, a modification of the surface and UV region of the spectra is observed with signs of charge carrier recombination rate acceleration. It is shown that the catalytic action of ZnO powders in polyethylene thermo-oxidation processes is determined by a combination of factors. In addition to dispersity and concentration, which are the key parameters, the morphology of ZnO particles, the presence of impurities, the surface state, and the distribution of active sites have a significant influence on catalysis. It has been experimentally demonstrated that these secondary factors can markedly affect the rate of radical formation in polyethylene films and alter their resistance to oxidation. ZnO nanoparticles exhibited low catalytic activity in both photocatalysis (rate constant 0.146 min^−1^) and thermocatalysis due to the high defect density of the crystallites and the presence of carbon-containing impurities. Submicron ZnO particles, owing to a high carrier generation rate and suppressed recombination (via trapping), demonstrated the highest photoactivity (rate constant 0.729 min^−1^). Submicron ZnO particles exhibit a catalytic effect on the thermo-oxidation of polyethylene (PE films); however, at concentrations above 8 wt.% a transition to an inhibiting effect is observed. ZnO microparticles catalyzed the oxidation of PE films over a broader concentration range (1–12 wt.%), with oxidation inhibition observed only at 18 wt.%. At the same time, they demonstrated moderate photocatalytic activity (rate constant 0.256 min^−1^). These characteristics of the samples correlate with data obtained by microscopy, photoluminescence spectroscopy, and X-ray diffraction analysis.

## 1. Introduction

Zinc oxide (ZnO) is a cost-effective and efficient catalyst widely used in processes aimed at the deep purification of environmental media from anthropogenic pollutants [1,2,3,4,5]. Along with TiO_2_, it is one of the most studied catalysts, and the choice between them is determined by the requirements for efficiency, stability, selectivity, and ecological safety of the process. ZnO surpasses TiO_2_ in the organic colorants degradation rate [6,7,8,9]; however, it falls behind in mineralization efficiency [7]. For selective oxidation with the retention of the carbon chain, ZnO is more preferable than TiO_2_ [10]. From the perspective of operational parameters, TiO_2_ surpasses ZnO in photochemical stability [11,12]. The main role in ZnO catalytic activity is played by intrinsic defects, such as oxygen vacancies, zinc vacancies, and interstitial zinc [13], as well as the technological possibility for varying the defect concentration is present [14]. Consequently, the investigation of the catalytic activity of ZnO particles, its enhancement, and achieving operational stability are of scientific and practical interest.

Most charge carriers involved in the formation of reactive oxygen species (ROS) (O_2_^•–^, ^1^O_2_, OH^•^) are generated upon photon absorption with energies exceeding the band gap width. However, at high temperatures, even in the absence of illumination, the concentration of charge carriers can be sufficient to initiate redox reactions on the ZnO surface. Therefore, the catalytic activity of zinc oxide in oxidation reactions manifests not only under photo-oxidative conditions, but also in the dark due to thermal activation.

The catalytic effect largely depends on the interfacial contact: the higher this value, the greater the number of accessible active sites, and, consequently, the course of catalytic reactions [15]. ZnO powder particles exhibit a wide variety of morphological forms, including nanorods, multipods, etc. Among them, ZnO multipods are considered the most promising photocatalysts [16] due to their hierarchical architecture. Owing to their spatial structure, the active surface area of ZnO multipods can reach values as high as 82 m^2^/g [17]. The high mean surface area of such structures promotes the enhancement of the interfacial contact area.

Another important manifestation of ZnO catalytic activity is the thermal-oxidative polymer destruction. The catalytic oxidation of organic substances involves their structural decomposition, which is particularly important for polymer disposal due to the problem of polymer waste accumulation [18].

Polyethylene (PE) remains one of the most widely used polymer materials owing to its low cost, chemical inertness, and ease of processing. The oxidation of PE is a complex radical chain process comprising the stages of initiation, chain propagation, branching, and chain scission. In this context, hydroperoxides (POOH), which are the main intermediate products of oxidation, play a crucial role, as their homolytic decomposition significantly increases the oxidation rate [19,20].

In industrial applications, pure PE is nowadays rarely used; instead, PE-based composite materials containing various inorganic fillers, such as metal oxides including ZnO, are more common. ZnO is typically employed as a white pigment, antistatic additive, UV-shielding agent, or antibacterial filler [21,22]. However, ZnO can also actively influence polymer oxidation processes under photo- or thermal exposure [23].

In contrast to ZnO photocatalysis, the mechanism of its catalytic effect under thermo-oxidative conditions remains under debate. In particular, the involvement of thermally generated electron–hole pairs has not yet been conclusively demonstrated experimentally. Several possible mechanisms of the thermocatalytic activity of ZnO have been proposed in the literature.

1. *Adsorption and activation of oxygen on surface defects.* The ZnO surface contains oxygen vacancies as well as Zn^2+^ and O^2−^ ions. Upon heating, the adsorption of molecular oxygen at defect sites, accompanied by the trapping of conduction-band electrons, is enhanced:

O_2_ + e^−^ → O_2_^•−^, (1)

The formed superoxide-anion can be further converted into other ROS:

O_2_^•−^+ e^−^ → O_2_^2−^, (2)

O_2_^•−^ + ^•^OH → ^1^O_2_ + OH^−^ (3)

Thus, under heating conditions, the ZnO surface may contain ROS, including O_2_^•−^, ^1^O_2_, and O_2_^2−^, which are capable of initiating radical processes. In [24], electron paramagnetic resonance measurements revealed a signal assigned to O^–^ on the ZnO surface with a *g*-factor of 2.014, the intensity of which increased with increasing temperature. According to DFT calculations [25], the adsorption energy and mechanism of atomic and molecular oxygen on the ZnO surface are determined by the crystallographic face and its atomic termination. On non-polar ZnO surfaces, the adsorption rates of O and O_2_ are low, whereas the dissociation of molecular oxygen is associated with a pronounced energy barrier. In contrast, on Zn-terminated polar surfaces, molecular oxygen can undergo dissociative adsorption with a negligible activation barrier.

2. *Catalysis of hydroperoxide (POOH) decomposition*. It is believed that the catalytic effect of ZnO under thermo-oxidative conditions is determined not primarily by the initiation of new radical formation, but rather by the acceleration of the decomposition of pre-existing polymer hydroperoxides [26]. In this case, the ZnO surface acts as a heterogeneous catalyst for POOH decomposition, facilitating the formation of alkoxyl (PO^•^) and hydroxyl (^•^OH) radicals, which accelerate the oxidation chain reactions:

POOH (ads.) → PO^•^ + ^•^OH (4)

Such a mechanism is widely discussed for metal oxides (including ZnO) and is considered one of the most plausible mechanisms of their involvement in the thermo-oxidative degradation of polymers [26]. The assumption that such a mechanism operates is consistent with the accelerated accumulation of carbonyl compounds in PE films observed in this work, as their formation is associated with hydroperoxide decomposition and subsequent oxidative transformations.

3. *Activation of C–H bonds by ZnO main oxygen sites*. According to this hypothesis, the main role is played by the main oxygen sites on the ZnO surface, which are capable of activating C–H bonds in PE macromolecules and promoting dehydrogenation reactions. Direct evidence supporting this hypothesis was provided in [27]. Using ab initio calculations, it was shown that the Gibbs free energy change for C–H bond dissociation at an oxygen site on the ZnO surface decreases from 236.8 to 1 kJ/mol due to the structural instability of this site, enabling continuous C–H bond dissociation during the reaction. The authors presented the following scheme of elementary steps [27]:

ZnO + –CH_2_–CH_2_– → ZnO–H + –CH^•^–CH_2_– (5)

ZnO + –CH^•^–CH_2_– → ZnO–H + –CH=CH– (6)

The presented mechanisms are not mutually exclusive and can operate simultaneously. It should also be noted that during catalysis, zinc carboxylates are formed on the ZnO surface, which shield the ZnO surface and, therefore, alter the catalytic activity of the particle surface [28].

However, the result of ZnO influence on the oxidation process may also be opposite. For example, it has been reported [29] that ZnO nanoparticles increase the thermal stability of polymers by raising the activation energy of thermal degradation. The obtained result was attributed to an increased probability of radical recombination on the surface of ZnO nanoparticles at higher radical concentrations, which results in a reduced oxidation rate. In addition, catalyst poisoning is possible, i.e., irreversible deactivation of active sites due to the adsorption of degradation products or impurities. At high filler concentrations, oxygen diffusion to the reaction zone is hindered. This effect can be particularly pronounced in agglomerated systems, where interparticle regions exhibit limited oxygen permeability. As a result, the oxygen supply rate to the active oxidation sites of polymer chains decreases, which can lead to the deceleration of thermo-oxidation processes and the manifestation of an inhibitory effect of the filler. Similar concentration-dependent effects have been reported for other metal oxides involved in radical processes [30]. Thus, such duality—the manifestation of both catalytic and inhibitory effects—is characteristic not only of ZnO but of most heterogeneous catalysts involved in radical reactions. Therefore, when analyzing the influence of nanoparticles on oxidation processes, it is necessary to consider not only their dispersion (specific surface area), but also their concentration.

Overall, the search for efficient photocatalysts is complicated by the need to perform long-term measurements using various types of equipment, as well as by the requirement for multiple sample manipulations. Typically, the sample is immersed in a solution containing the target pollutant and exposed for an extended period of time to an appropriate light source; subsequently, the solution with the target pollutant is monitored over time to evaluate the stages of decomposition. The identification of efficient thermo-oxidative catalysts is even more challenging, as these processes occur in the solid phase, where diffusion rates are reduced.

The effectiveness of ZnO as a catalyst directly depends on the nature and concentration of defects. In this regard, we consider photoluminescence (PL) spectroscopy to be a highly promising and rapid method for evaluating the catalytic activity of ZnO-based photocatalysts. ZnO is characterized by an intense and well-defined luminescence spectrum with characteristic bands in the UV and green regions of the light spectrum [31]. Ultraviolet (UV) luminescence is related to the radiative recombination of free or bound excitons occurring in regions near the edge of the semiconductor band gap. Green (GL) luminescence in zinc oxide originates from the radiative recombination of electrons at intrinsic defects in the crystal lattice. The intensity ratio of these emission bands is, in turn, related to the crystalline quality of ZnO samples.

Time-resolved PL spectroscopy measurements allow the evaluation of the kinetic characteristics of luminescence bands associated with the intrinsic defects of the crystal lattice, which act as charge carrier traps. It should be noted that although the PL spectroscopy method does not provide quantitative information, compared to structural analysis techniques, it effectively complements them by specifying the role of point defects in the crystal lattice.

Thus, catalysis and photo-stimulated processes, although chemical and physical in nature, share similar stages associated with charge carrier generation and diffusion within the ZnO crystal. The presence of deep charge carrier trap states can significantly influence both the catalytic activity and the luminescence yield of ZnO samples.

In the present work, for the first time, a comprehensive analysis of the relationships between the photocatalytic, thermo-oxidative, and photoluminescence properties of ensembles of ZnO nano- and microstructures is presented, together with an investigation of the correlation between X-ray diffraction (XRD) data and microscopic observations.

## 2. Results and Discussion

### 2.1. Study of Morphology and Structural-Phase Compositions of ZnO Samples

**Figure 1:**
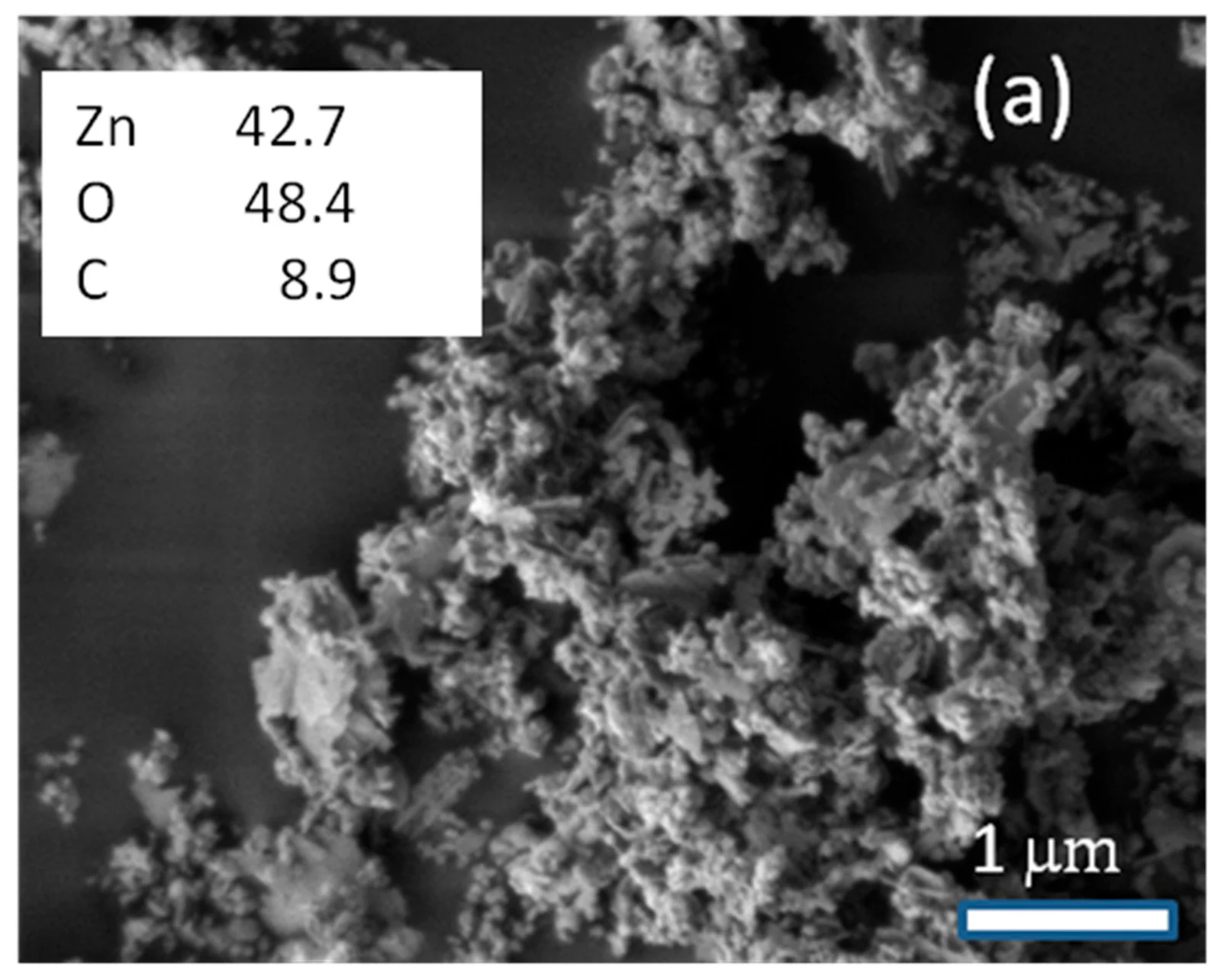

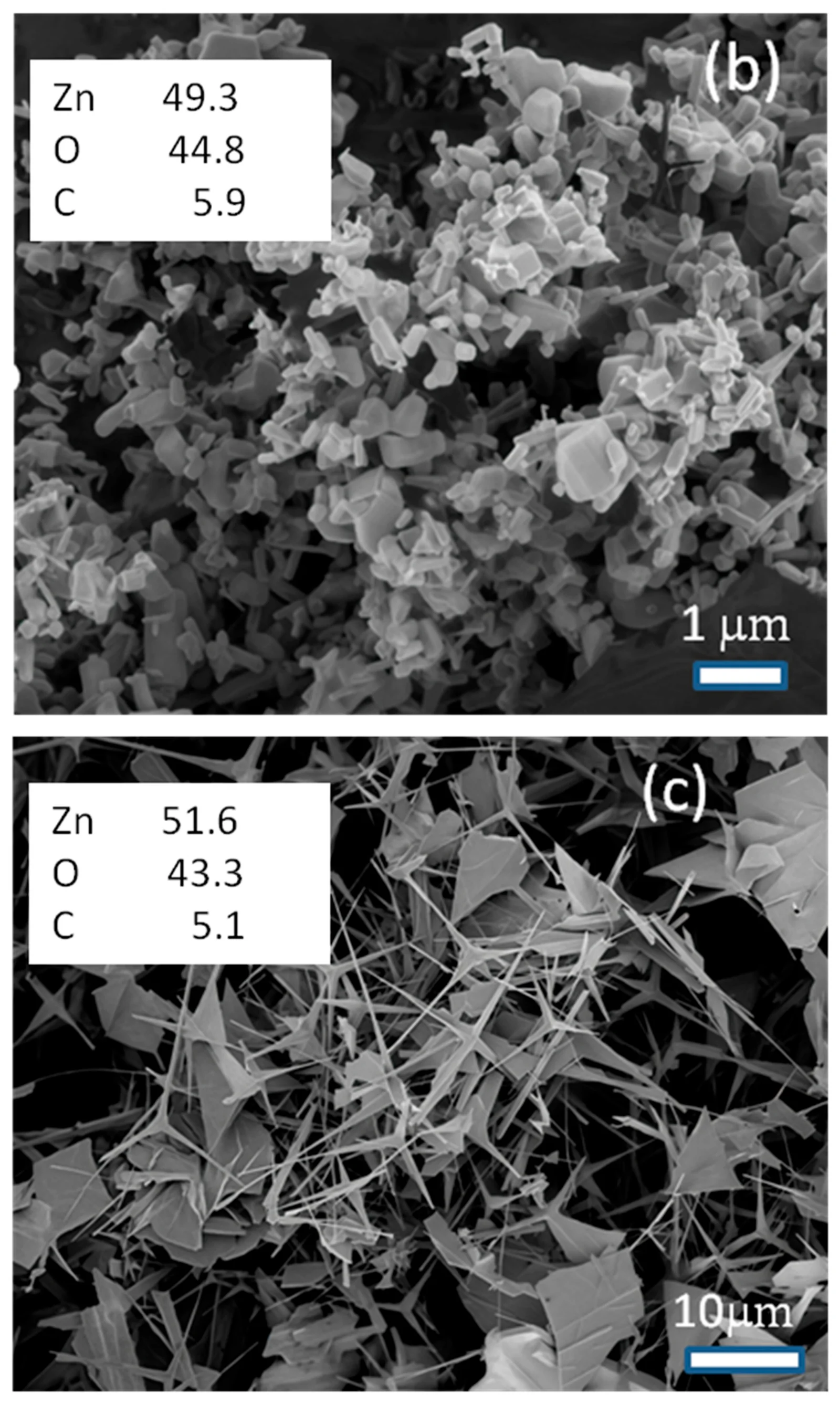
SEM images of ZnO samples: ZnO-1 (**a**); ZnO-2 (**b**); ZnO-3 (**c**). Insert: the atomic composition of the powders according to EDX data.

According to the scanning electron microscopy (SEM) data (Figure 1a), ZnO-1 nanoparticles exhibit a predominantly spherical morphology with an average size in the range of 30–50 nm. The submicron ZnO-2 particles (Figure 1b) are well-defined crystalline structures with sizes ranging from 100 to 500 nm.

The highest morphological diversity was observed for ZnO-3 microparticles (Figure 1c), synthesized using microwave plasma. Specifically, three distinct types of microparticles were observed: rod-like structures with lengths of up to several tens of microns and diameters of up to 200 nm; tetrapods with “limb” lengths of up to several tens of microns and diameters of up to 400 nm in the core region; and plate-like structures with lateral dimensions of up to several tens of microns and thicknesses of up to 400 nm. According to the EDX data, the ZnO-2 and ZnO-3 samples exhibited an excess of Zn and background carbon contamination. In contrast, the ZnO-1 sample showed an increased content of presumably absorbed oxygen and carbon contaminants. The mean specific surface area values for the ZnO-1, ZnO-2, and ZnO-3 samples were 32 m^2^/g, 3.8 m^2^/g, and 2.6 m^2^/g, respectively.

**Figure 2:**
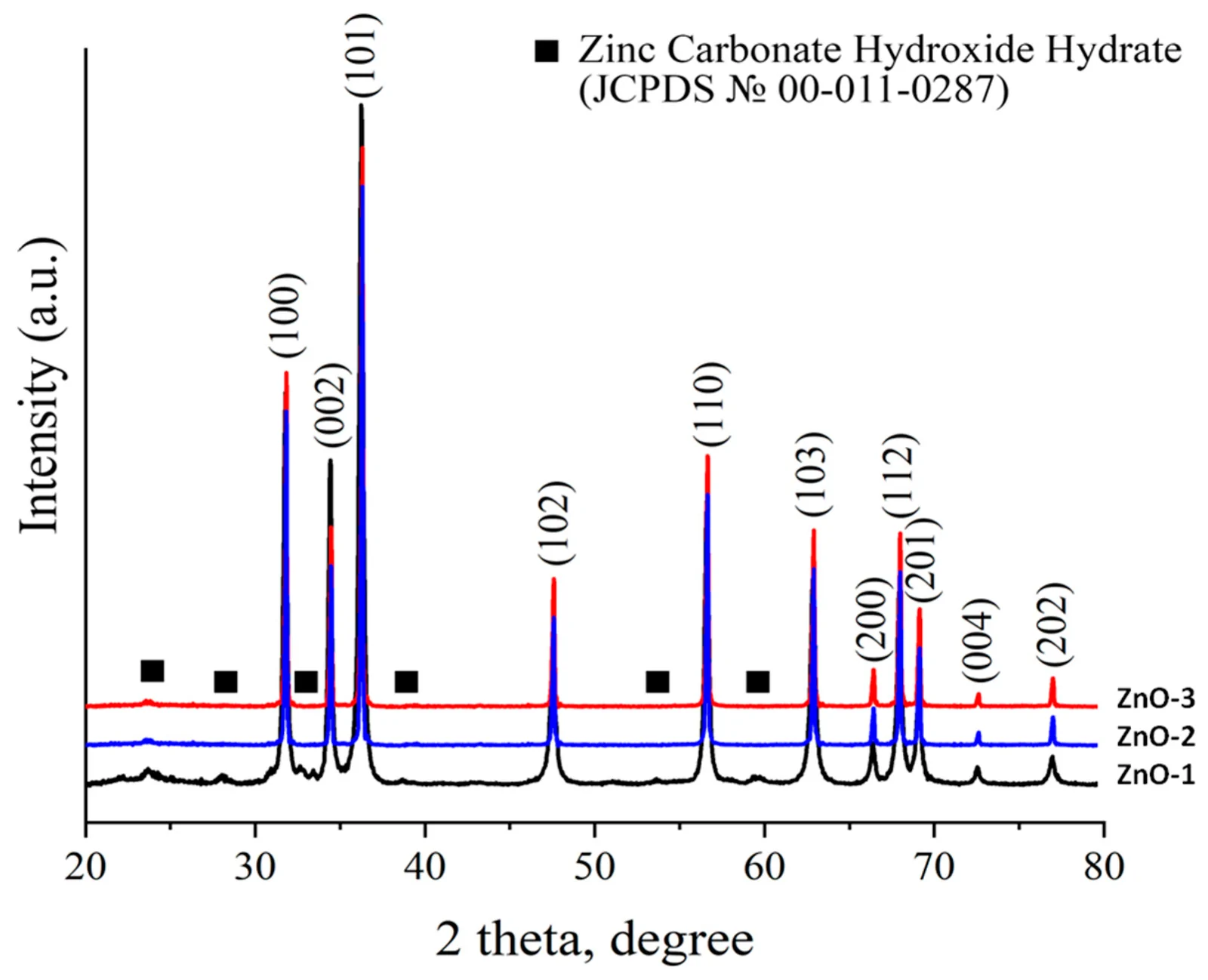
XRD patterns of ZnO nanoparticles of different types.

The observed diffraction peaks (Figure 2) for samples of all types correspond to the typical hexagonal wurtzite-type ZnO structure (Reference from the JCPDS database No. 00-036-1451), belonging to the space group P6_3_mc [32,33]. The X-ray diffraction pattern of sample type 1 contains additional peaks that are associated with the zinc hydroxycarbonate phase (JCPDS reference: No. 00-011-0287), which is an intermediate product in the synthesis, indicating incomplete decomposition of the precursor/intermediate phase. No impurity phases were detected in the X-ray diffraction patterns of sample types 2 and 3.

The lattice parameters of the identified ZnO phase are determined as follows:

(7) $$\frac{1}{{d_{hkl}}^{2}}=\frac{4}{3}\cdot \frac{\left(h^{2}+k^{2}+h\cdot k\right)}{a^{2}}+\frac{l^{2}}{c^{2}}$$

where *a*, *c*—lattice constants, Ǻ; *h*, *k*, *l*—the Miller indices; *d_hkl_*—the interplanar distance for a specific plane, Ǻ.

The size of the coherent scattering regions, as well as the microstrain values, were calculated using the Williamson–Hall model [34,35]:

(8) $$\beta \cdot cos\theta =\frac{k\cdot \lambda }{D}+4\cdot \varepsilon \cdot sin\theta$$

where *β* is the total peak broadening, rad.; *λ* is the wavelength of X-ray radiation, nm; *D* is the crystallite size (coherent scattering region), nm; *θ* is the center of gravity of the peak, rad.; *k* = 0.9 is the coefficient representing the shape factor of a sphere [36].

Total broadening includes broadenings due to coherent scattering regions, microstrain values, and instrumental broadening of the device. The latter was taken into account at the beginning of the analysis. Instrumental broadening was subtracted using:

(9) $$\beta =\sqrt{\beta_{meas}^{2}-\beta_{inst}^{2}}$$

where *β_inst_* was determined using a standard reference sample.

The calculated values of the crystallite size associated with ZnO samples of different types, determined from the Williamson–Hall model, were estimated from the plots *β* cos *θ* versus 4 sin *θ* and presented in Table 1.

The lattice parameters obtained for the ZnO nanoparticles of different types are in good agreement with those reported for the hexagonal wurtzite etalon structure (e.g., a = 3.249 Å, c = 5.207 Å). The minor observed discrepancies may be associated with factors such as the presence of internal defects in the ZnO structure (e.g., oxygen vacancies, zinc vacancies, or interstitials), as well as differences in the methods used to produce the nanoparticles. Significant differences are observed in the sizes of the coherent scattering regions of the nanoparticles, as well as in the microstrain values. In type 1 samples, the microstrain values are one order of magnitude higher than those observed in type 2 and 3 samples. The sizes of the coherent scattering regions are also approximately half as large and are comparable to the particle sizes estimated from SEM observations.

### 2.2. Investigation of Photoluminescence Properties of ZnO Powders

**Figure 3:**
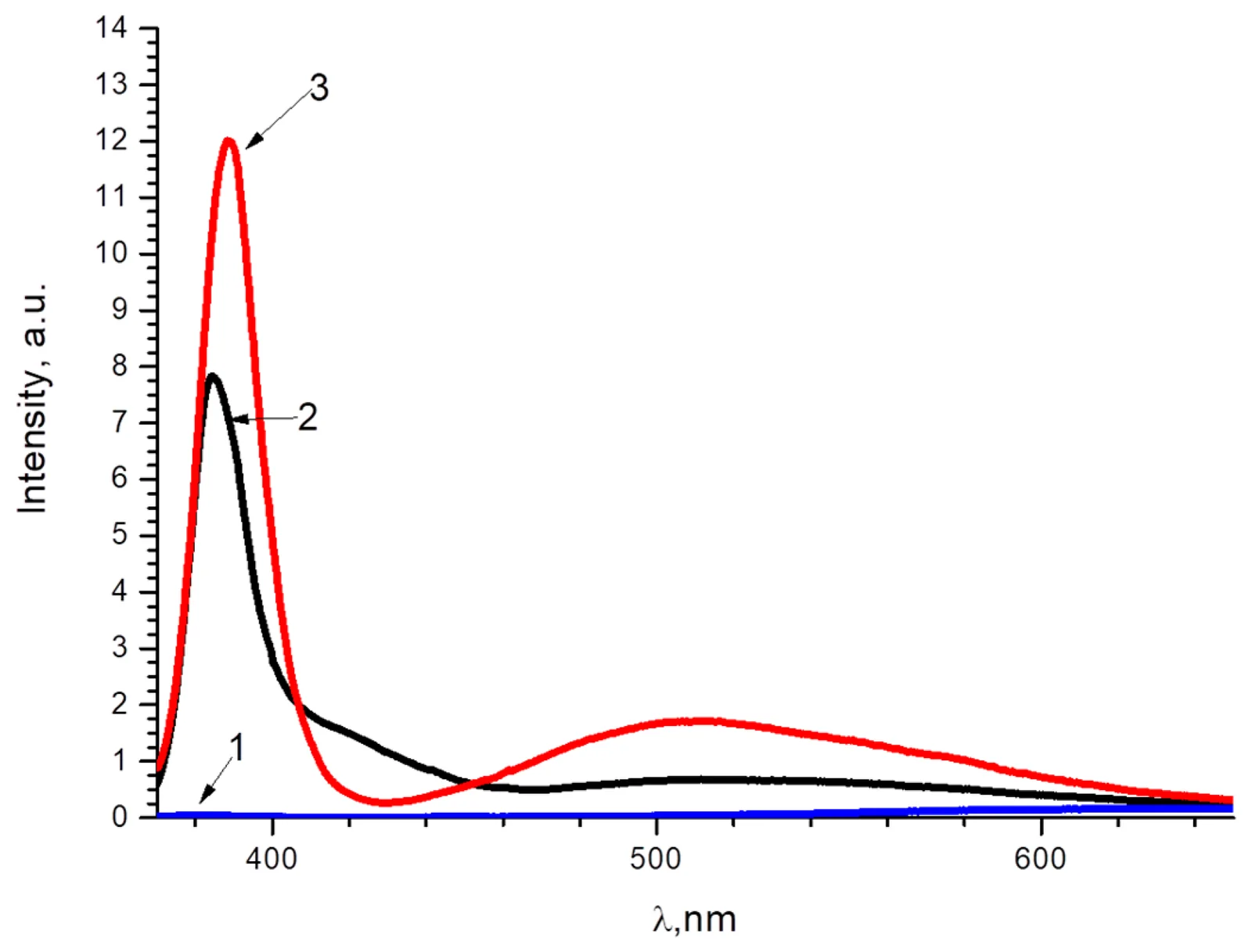
PL spectra of ZnO samples (excitation at λ = 315 nm): ZnO-1 (curve 1); ZnO-2 (curve 2); ZnO-3 (curve 3).

At the initial stage, panoramic PL spectra of ZnO particles were analyzed (Figure 3).

It can be seen that, in powders with different dispersities, two distinct luminescence regions are observed at room temperature upon excitation in the interband transition region: near-band-edge ultraviolet (UVL) emission near the absorption edge in the range of 380–390 nm and a non-elementary long-wavelength green luminescence band (GL), determined by the presence of defects and/or impurities [37,38,39,40,41]. The near-band-edge short-wavelength luminescence, as demonstrated in low-temperature studies, is of excitonic origin [37,41]. At low temperatures, the luminescence band consists of several Wannier–Mott exciton-related emission components, including free excitons, donor–acceptor-bound excitons, and their phonon replicas. Due to the high exciton binding energy (60 meV), the observation of intense excitonic emission in ZnO is possible even at room temperature [37]. The maximum location, shape, and intensity of the short-wavelength band in ZnO at room temperature depend on the degree of defectiveness, dispersity, and gas medium during synthesis, and possibly are defined by the superposition of contributions of free, bound, and phonon-replicated excitons, and are shifted in different samples in a quite wide wavelength range from 375 to 395 nm. The non-elementary long-wavelength luminescence of ZnO can be associated with various intrinsic defects and impurities. Virtually all types of intrinsic defects have been considered as possible structural sources of long-wavelength luminescence, including zinc vacancies (V_Zn_), oxygen vacancies (V_O_), zinc interstitials (Zn_i_), oxygen interstitials (O_i_), and their complexes [37,38,39,40,41]. GL is sensitive to the stoichiometry, and a conclusion that oxygen vacancies are responsible for this luminescence has been drawn by a number of authors [42]. In principle, oxygen vacancies can be located both in the bulk volume and on the surface of ZnO in three different charge states, including neutral V_O_^0^ (the properties of neutral oxygen vacancies in ZnO are analogous to those of F-centers, which are well studied in wide-band-gap ionic oxides such as CaO, SrO, BaO, MgO, and Al_2_O_3_), singly ionized V_O_^+^, and doubly ionized V_O_^++^. In the bulk of ZnO, the most stable intrinsic growth defect (a deep donor), according to quantum-mechanical calculations, can be an oxygen vacancy that traps two electrons, V_O_^0^, which is analogous to an F-center. Undoped ZnO samples are n-type at room temperature due to the predominance of donor defects (V_O_^0^, Zn_i_^0^) in the crystal lattice. Unlike V_O_^0^, Zn_i_^0^ is a shallow donor, presumably determining the n-type conductivity of undoped ZnO at room temperature. A deviation from stoichiometry toward an increased Zn content relative to O leads to an increase in long-wavelength luminescence, i.e., to a decrease in the I_UVL_/I_GL_ intensity ratio.

**Figure 4:**
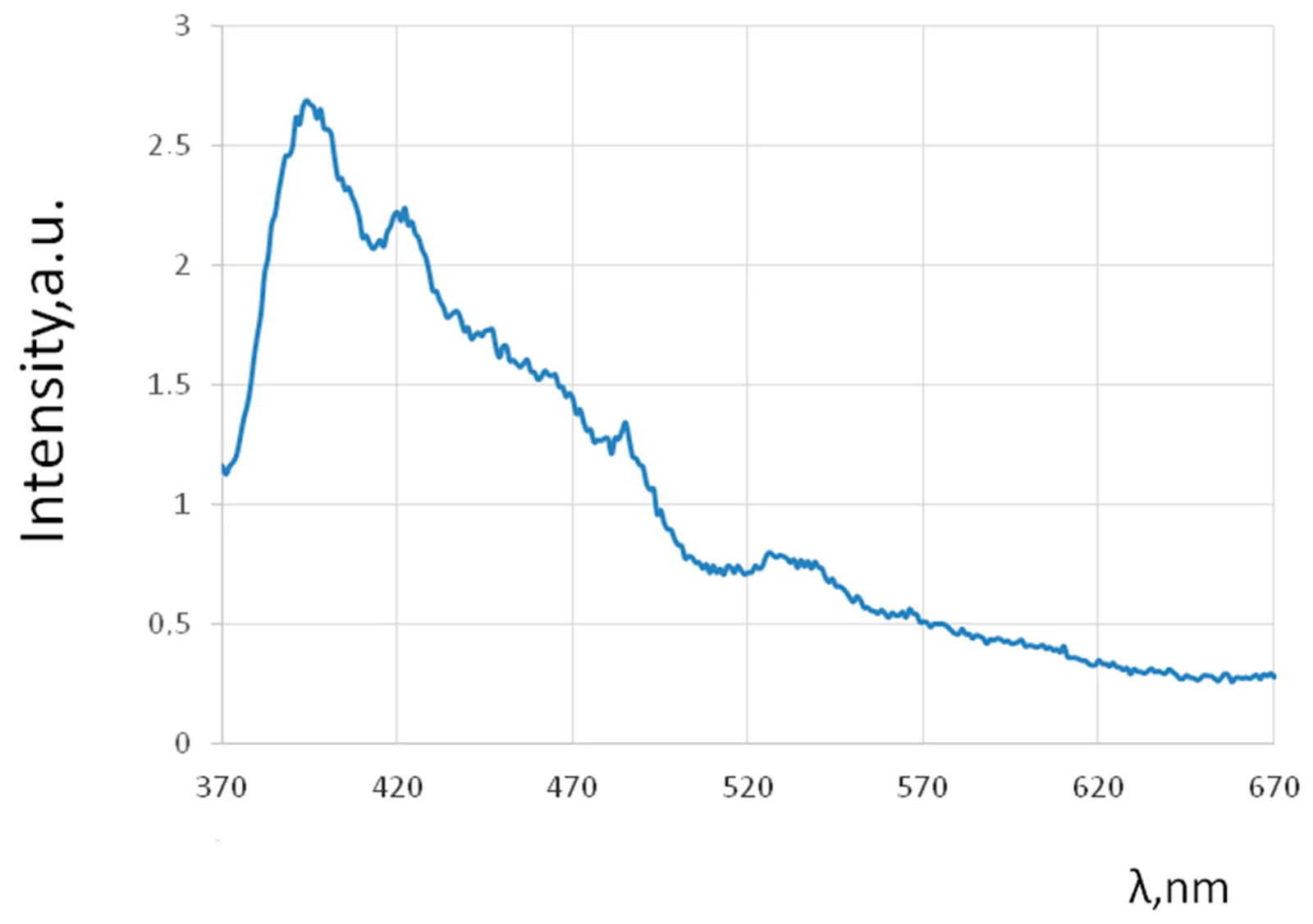
Photoluminescence spectrum of nanosized ZnO-1 powder from Figure 3 on an enlarged scale.

The overall luminescence intensity of the nanosized ZnO-1 powder is lower compared to that of the other samples. Figure 4 presents the PL spectra of the nanosized ZnO-1 powder on an enlarged scale.

It is noteworthy that the introduction of organic and metallic impurities into ZnO micropowders leads to a decrease in luminescence intensity at ~3.2 eV (384–387 nm) and to a change in the I_UVL_/I_GL_ intensity ratio, accompanied by modifications of the structured long-wavelength luminescence.

It is known that a space charge region is formed in the near-surface layer of an n-type semiconductor, with a charge opposite to that of the surface and compensating for it. This leads, due to upward band bending, to a difference in the ratio and types of defects between the surface and the bulk of the semiconductor, which is controlled by the impurity and defect energy levels, the band bending depth, and the width of the depletion layer. The volume-to-surface ratio in nanodispersed powders differs significantly from that in micropowders, and the contribution of surface luminescence becomes dominant. The luminescence maximum of the ZnO-1 nanopowder, similar to that of bulk micropowder samples and corresponding to near-band-edge luminescence, is located in the UV region (398 nm) and is presumably associated with excitonic emission. The emission at 423 nm is characteristic of ZnO nanoparticles modified with carbon [43]. The presence of carbon-containing components in the ZnO-1 sample was confirmed by XRD data. The low intensity and complexity of the PL spectrum of the ZnO-1 sample can be explained by the presence of carbon-containing impurities, high surface defect density, high dispersity of powder nanoparticles, and gas sorption on these surface sites. Presumably, the PL spectrum of the ZnO-1 nanopowder sample is mainly determined by surface luminescence, whereas excitonic and defect-related bulk luminescence are suppressed, resulting in a decrease in PL intensity. The surface state plays a significant role in the formation of the overall luminescence signal, which is determined by contributions from both surface and bulk processes. This allows photoluminescence spectroscopy to be employed as an effective method for studying the photocatalytic and thermo-oxidative characteristics of ZnO. The decrease in ZnO particle size affects its emission properties and photocatalytic activity. ZnO-1 is one of the most studied nanomaterials, and a range of recent studies has been devoted to investigating the relationship between ZnO nanoparticle size and its photoluminescent, photocatalytic, and other properties, as well as to analyzing the origin of new electronic, absorption, and emission properties in nanosized ZnO systems [44].

The photogenerated electrons and holes produced upon photoexcitation in the band-to-band transition region stimulate various redox reactions on the surface, leading to changes in the electrophysical and optical characteristics of the sample [45]. At a high surface-to-volume ratio, the surface state, determined on the one hand by adsorbed species and on the other hand by their photodesorption, significantly affects the course of optical and electronic processes [45]. The photodesorption of oxygen, N_2_, and other molecules from the ZnO surface at various temperatures and under different illumination conditions has been confirmed by direct mass spectrometry studies. Moreover, at high light flux densities in the self-absorption region, not only adsorbed oxygen but also surface lattice anions can be desorbed [45]. The increase in the contribution of “excitonic luminescence” induced by annealing in a hydrogen and carbon monoxide atmosphere is neutralized upon oxygen adsorption at room temperature, when oxygen diffusion can be neglected. The formation of positively charged oxygen vacancies on the surface leads to an increase in the intensity of excitonic PL, whereas oxygen addition restores the spectrum to its initial state.

The component separation of the long-wavelength ZnO luminescence can be effectively achieved by varying the luminescence registration delay time after the excitation pulse.

**Figure 5:**
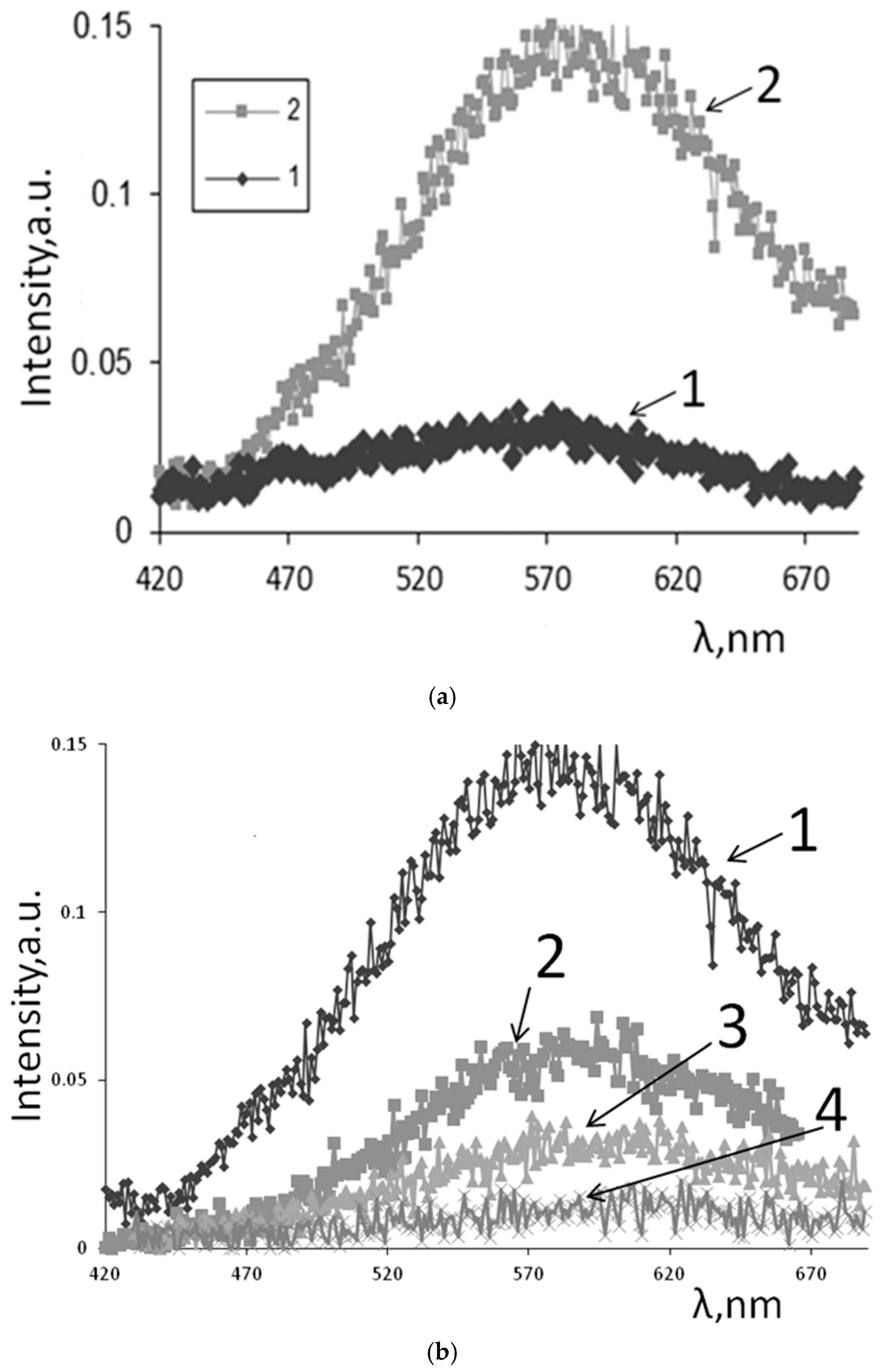
PL spectra of ZnO-1 nanopowder under excitation at λ = 355 nm (3.5 eV) at various strobe delays and durations: (**a**) delay of 20 μs with strobe durations of 5.7 μs (1) and 1000 μs (2); (**b**) strobe duration of 1000 μs with delays of 20 μs (1), 70 μs (2), 150 μs (3), and 500 μs (4).

Figure 5 represents the long-wavelength PL spectra of the ZnO-1 nanopowder recorded at different delay times and luminescence registration durations following an excitation pulse. At long registration times, an intense emission band with an effective maximum at ~570 nm is observed. Accordingly, long-term luminescence components with decay times starting from τ ~300 μs are detected. Components in the millisecond time range cannot be reliably resolved, likely due to the low luminescence intensity of nanopowders. The long-term emission band at 460 nm, characteristic of the other samples and attributed to a triplet transition associated with F-centers, is not observed for nanopowder. The observed long-wavelength luminescence is therefore likely of impurity-related origin, which is consistent with the XRD data.

**Figure 6:**
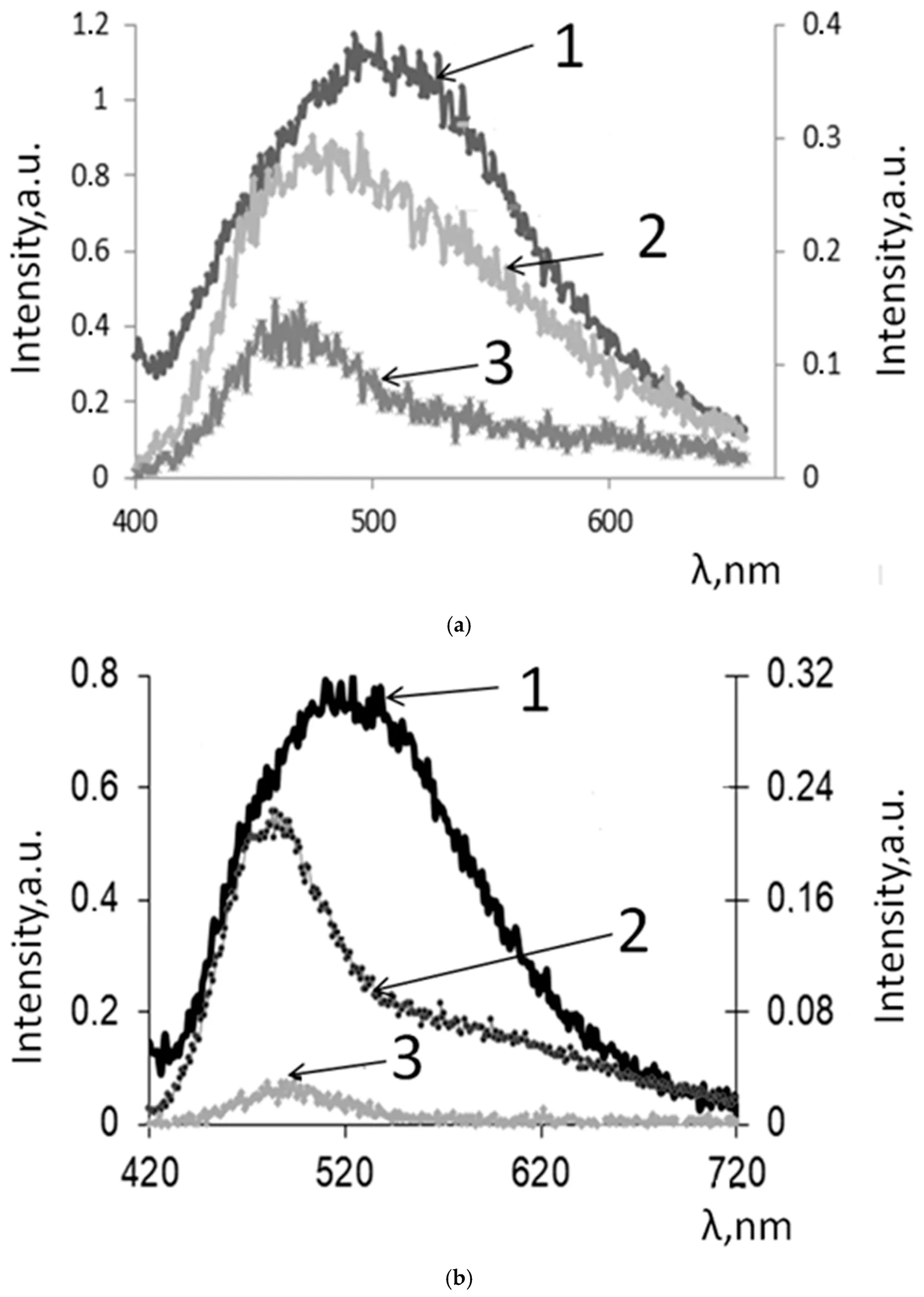
PL spectra of the ZnO-2 submicron micropowder obtained at different strobe delays (indicated in the legends) with a strobe duration of 1000 μs: (**a**) excitation at λ = 355 nm (3.5 eV) with delays of 15 μs (1-left axis), 30 μs (2-right axis), 70 μs (3-right axis); (**b**) excitation at “excitonic” band at 380 nm (3.26 eV) with delays of 20 μs (1-left axis), 70 μs (2-right axis), 1000 μs (3-right axis).

In submicron-sized powders, a broad, non-elementary long-wavelength luminescence band (Figure 6) consists of at least three overlapping bands with maxima at ~460, 490–500, and 530 nm with different decay characteristics. A weak broad emission extending from 560 to 700 nm is observed in the long-wavelength tail. A spectral feature in the region of ~425 nm is also observed in the spectra. The emission bands centered at 425 and 490 nm exhibit rapid decay, while the 530 nm band shows a decay time of ≤80 μs, and the weak long-wavelength emission exhibits a decay time of ~100 μs.

At long delay times and for increased strobe exposure durations (up to milliseconds), the photoluminescence spectra of micropowders demonstrate emission only in the region of 460 nm, which may originate from spin-forbidden triplet-to-singlet transitions of excited defect centers (F* and F+*).

The UV luminescence band is efficiently excited over a wide spectral range of interband transitions, with a pronounced maximum at 287 nm and a pronounced oscillatory structure. This behavior indicates weak localization of electronic states and a strong coupling with the surrounding crystal lattice. A similar excitation spectrum is observed for the blue–green luminescence band at ~490 nm, although the excitation efficiency is somewhat higher near the excitonic absorption band. The excitation maximum of the observed persistent luminescence bands coincides with the excitation maximum of the UV emission band, which may indicate their excitation due to self-absorption of UV radiation, i.e., due to the excitation of radiative annihilation of excitons localized at surface defects. The introduction of organic dopants into ZnO powders leads to a reduction in short-wavelength luminescence at 384–387 nm. At the same time, a change in the I_UVL_/I_GL_ intensity ratio is observed, accompanied by modifications in the long-wavelength structured luminescence.

**Figure 7:**
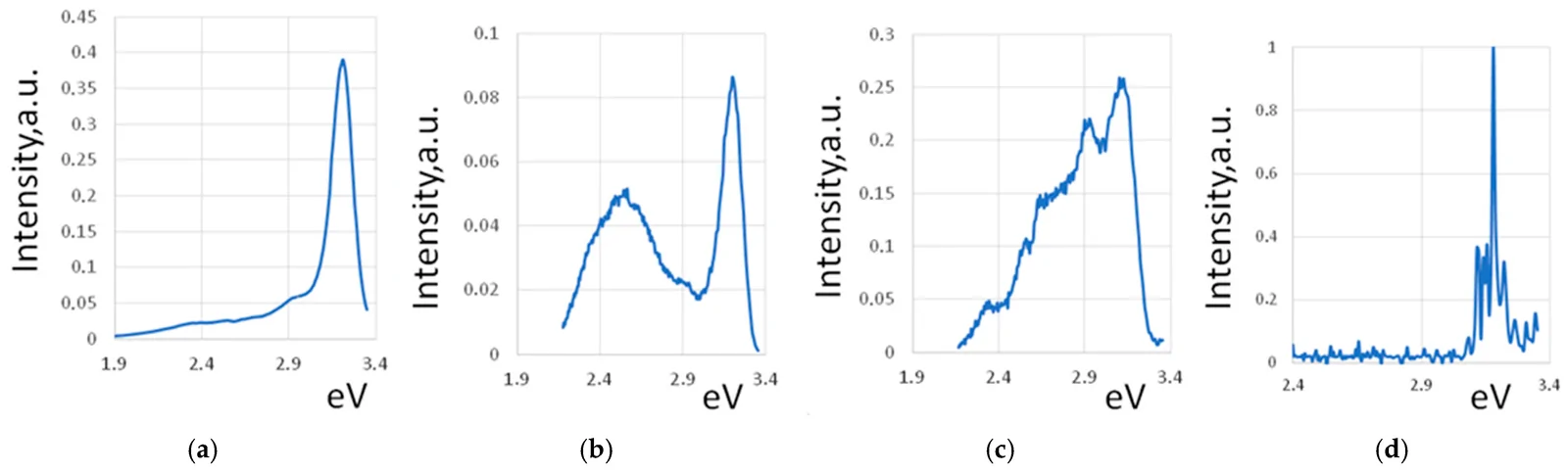
Normalized luminescence spectra of ZnO-2 powder recorded under excitation at λ = 355 nm and standard signal registration conditions, measured at excitation intensity (Io) change by four orders of magnitude: (**a**) maximum intensity Io = 1, used in all previous experiments; (**b**) 100-fold reduced intensity Io = 10^−2^; (**c**) 1000-fold reduced intensity Io = 10^−3^; (**d**) 10,000-fold reduced intensity Io = 10^−4^.

Upon varying the excitation density in ZnO-2 samples, a change in both the PL spectra and the luminescence yield is observed. These effects can be associated with the ratio between the number of generated photons and the concentration of bulk growth defects, impurities, and surface defects (Figure 7).

The yields of UV and long-wavelength luminescence exhibit different dependences on the excitation pulse intensity. The yield of long-wavelength luminescence shows only a weak dependence on excitation intensity. The growth of luminescence intensity at the onset of emission can be approximated by a power-law dependence with an exponent of 0.5 at low intensities and close to 1 at higher intensities.

The dependence of the exitonic luminescence yield is more complex. At the highest excitation intensities, a linear increase in yield (corresponding to a quadratic dependence on excitation intensity) is observed (Figure 7a). At very low intensities, several orders of magnitude lower than those typically employed in standard experiments, a structured surface emission with a quantum yield close to 1 is observed (Figure 7d). This emission decreases as the excitation pulse intensity increases. It may be associated with the dissipation of the excitation pulse and/or short-lived, intense luminescence of free excitons at surface defects (adsorbed gases).

Within this interpretation, the dissipation-related bands can be attributed to impurity states and appear in the spectral range from 380 to 460 nm, which is observed experimentally (Figure 7c). The conditions for luminescence registration in reflection geometry and the use of powders make spectra highly sensitive to surface conditions and to sorption–desorption of atmospheric gases.

At low excitation densities, the excitation process occurs only at the grain surface; consequently, the luminescence signal is mainly governed by dissipation on surface-sorbed gases.

**Figure 8:**
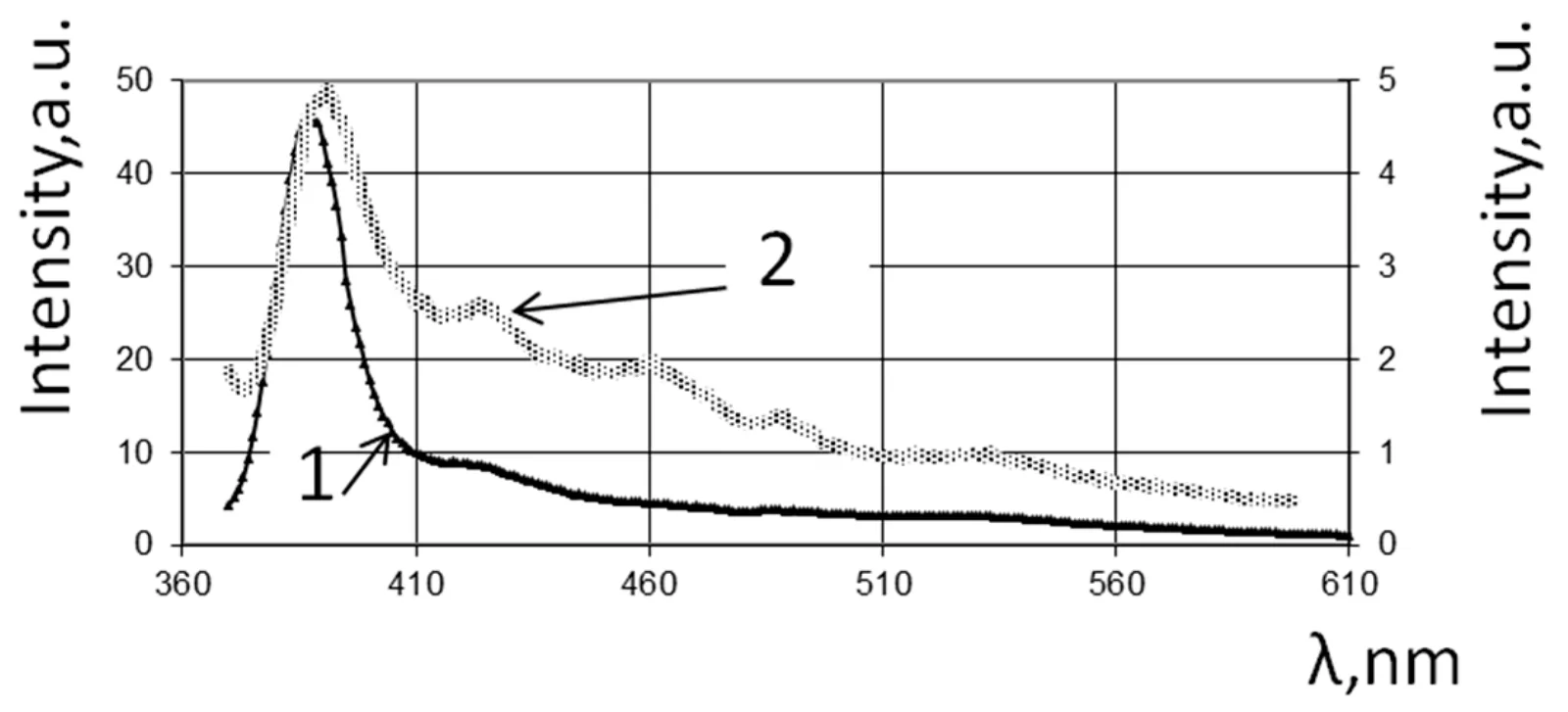
PL spectra of ZnO-2 (1) powder and organic-doped ZnO-2 (2—right axis) obtained with excitation λ = 255 nm and standard registration conditions.

The spectrum recorded at low excitation densities in Figure 7c is similar to that of the ZnO-1 nanopowder with the smallest particle size (Figure 4), which contains carbon-based impurities, as well as the spectrum of organically doped ZnO (Figure 8). Hence, the adsorbed carbon-based components are present in the near-surface layers even in high-purity ZnO-2 samples and determine their luminescence properties under low excitation intensities. Overall, it can be inferred that strong dissipation of the excitation light suppresses bulk excitation and excitonic luminescence, which undergoes self-absorption. As a result, an emission mostly associated with dissipation is observed.

**Figure 9:**
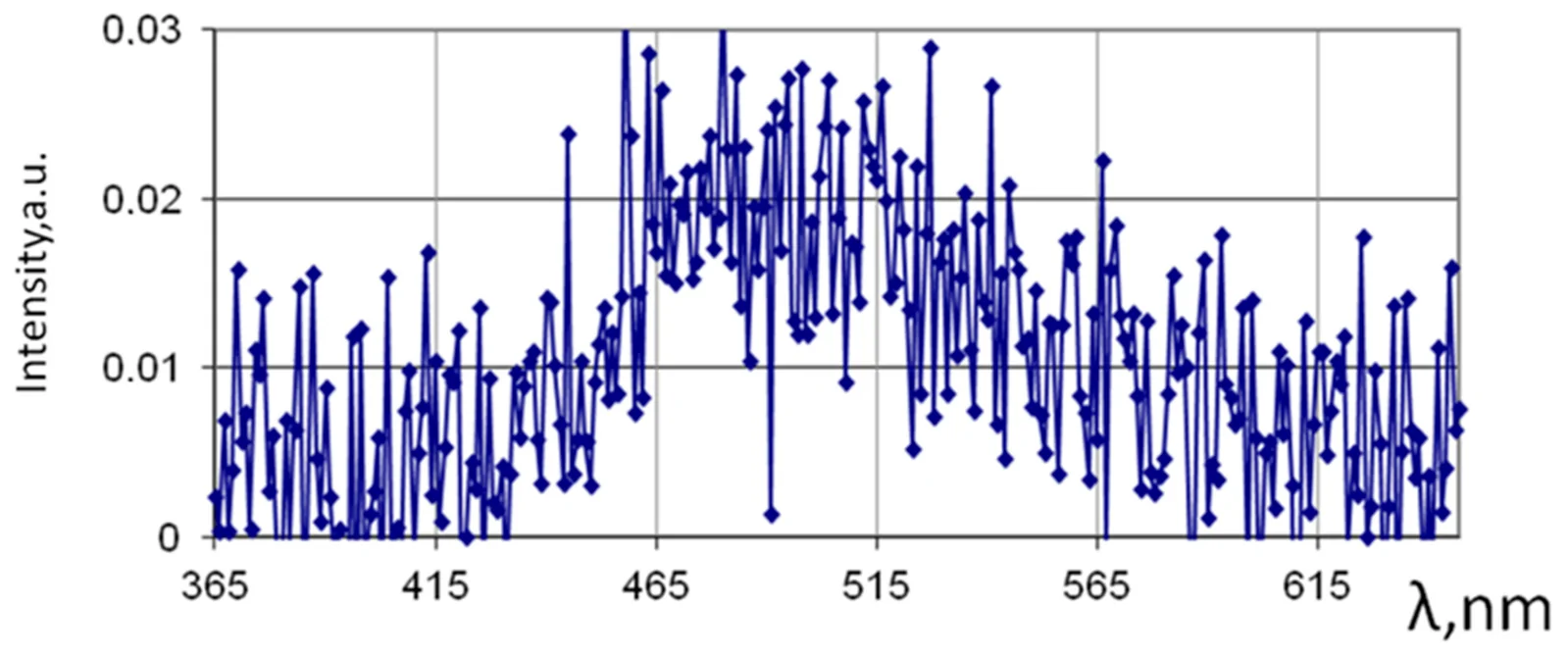
PL spectra of the micron-sized ZnO-3 powder recorded under excitation at λ = 355 nm, with a strobe delay of 20 μs and a strobe duration of 1000 μs.

The luminescence spectrum of the micrometer-sized ZnO-3 powder (Figure 9) closely resembles the luminescence spectrum of the submicron ZnO-2 powder. However, judging by the I_UVL_/I_GL_ ratio, it exhibits slightly greater nonstoichiometry with a predominance of donor centers (V_o_, Zn_i_). Long-lived stages are not observed at all, and/or the instrument sensitivity is insufficient to detect them. When the gating time is increased from 5.7 to 1000 µs, and the standard delay is 0.7 µs, the luminescence changes very little, whereas a delay of >20 µs leads to a complete absence of luminescence. Figure 9 shows the longest detectable luminescence of the micron-sized ZnO-3 powder, with a luminescence maximum at 490 nm and a decay duration of ~5.7 µs, i.e., matching the excitation pulse.

The majority of the experimental data can be consistently explained by assuming that the formation and preferential survival, under different synthesis conditions, of defects in the anion sublattice of real ZnO structures, which also determine the surface state, including the adsorption and desorption of atmospheric gases.

The broad UV emission bands observed in the 370–390 nm spectral range, registered depending on the excitation density, particle size and morphology, and other factors, are attributed not to the radiative recombination of unfree excitons, but rather to relaxed in the regular lattice autolocalized (ALE), or on the surface (ALEs), impurity, or defect band excitons.

According to both the literature and our experimental results, the neutral oxygen vacancy (F-center) in bulk ZnO represents a deep state with an energy depth of 0.64 eV relative to the valence band. The free electrons and F^+^, V_o_^++^ excited hole centers generated upon the excitation of F-centers lead to the recombinational formation of defect-bound excitons, or to the localization of free excitons and their radiative annihilation on defects. The luminescence of such localized excitons falls within spectral regions of higher optical transparency of the powder’s crystallites, thereby leading to the excitation of bulk impurity and structural defects, as well as the triplet state of the F-center, responsible for the long-lived emission at 460 nm.

At room temperature, the absorption coefficient of ZnO in the spectral region corresponding to the exciton absorption band and band excitation is 10^4^–10^5^ cm^−1^, which gives an estimated excitation depth of 0.1–1 μm. In contrast, at energies corresponding to the ALE annihilation (3.15–3.2 eV), the excitation depth value is 1–10 μm, leading to the excitation of bulk luminescence. The defectiveness of the crystal lattice and the presence of absorbing impurities in ZnO-1 nanoparticles lead to a decrease in their emission intensity.

### 2.3. Influence of ZnO Powder Morphology and Structure on the Thermo-Oxidation of PE Films

**Figure 10:**
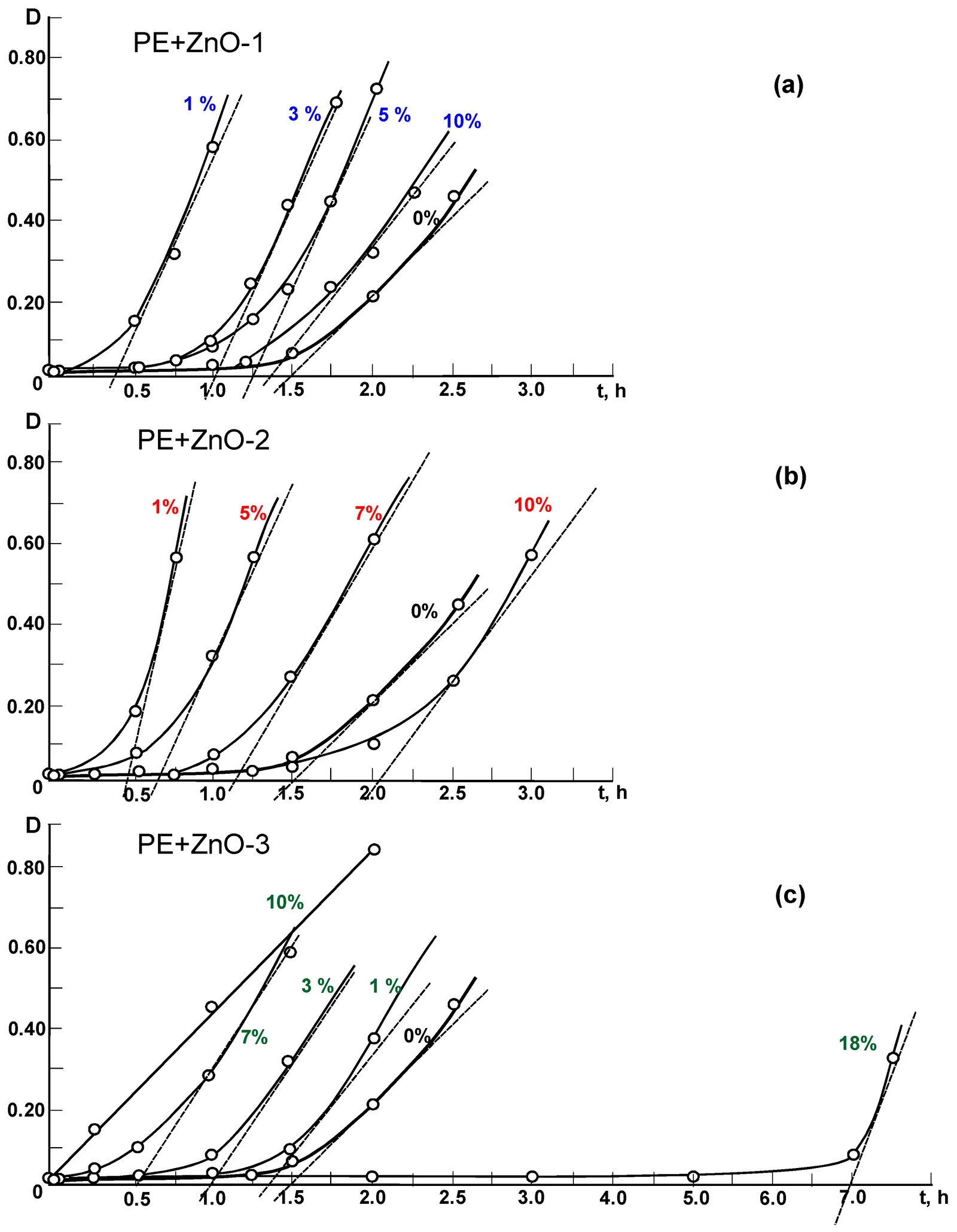
Accumulation of carbonyl groups in PE foams containing ZnO during thermo-oxidation: (**a**) ZnO-1, (**b**) ZnO-2, (**c**) ZnO-3 (powder concentrations are indicated on the curves).

In our experiment, the incorporation of ZnO powders into PE films led to a change in the duration of the oxidation induction period (OIP) (Figure 10). During the thermo-oxidation of filler-free PE films, the OIP is approximately 1.5 h. At low ZnO concentrations (1–7 wt.%), a reduction in the OIP of PE films is observed. In contrast, at high ZnO concentrations (≥10 wt.%), the OIP increases compared to the control film (Figure 10).

The dependences of the OIP on the concentrations of ZnO-1 and ZnO-2 are somewhat similar (Figure 10, curves 1, 2). Both powders produce the maximum reduction in the OIP of PE films at a concentration of 1 wt.% (Figure 10a,b). However, the particle size of ZnO-1 is approximately 10–15 times smaller than that of ZnO-2, indicating that the type II powder catalyzes the thermo-oxidation process of PE films more effectively.

The low catalytic activity of the ZnO-1 powder is related to a combination of several factors, including the particle morphology and the high defectiveness of the structure. According to the FL spectroscopy data (Figure 4 and Figure 7c), carbon-containing impurities are present in both ZnO-1 and ZnO-2 samples. In ZnO-1, they were introduced during synthesis and form a stable phase, whereas in ZnO-2 they are present only as surface contamination. The obtained results are consistent with the XRD data. Indeed, the ZnO-1 powder is characterized by low crystallite quality (Table 1, Figure 2 and Figure 3) and a significant content of carbon-containing impurities (Figure 2).

**Figure 11:**
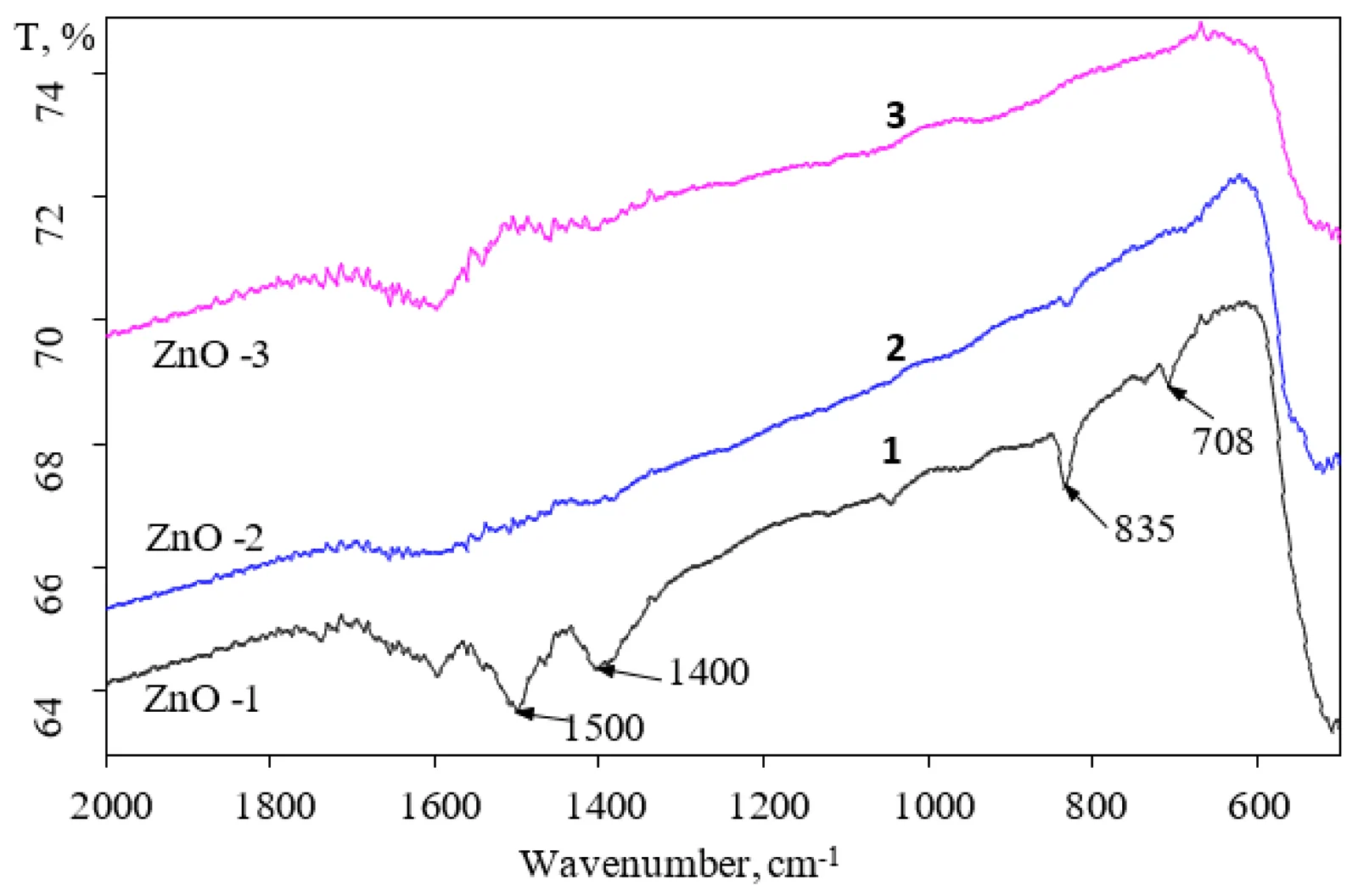
ATR-FTIR spectra of the studied zinc oxide powders: 1—ZnO-1; 2—ZnO-2; 3—ZnO-3.

**Figure 12:**
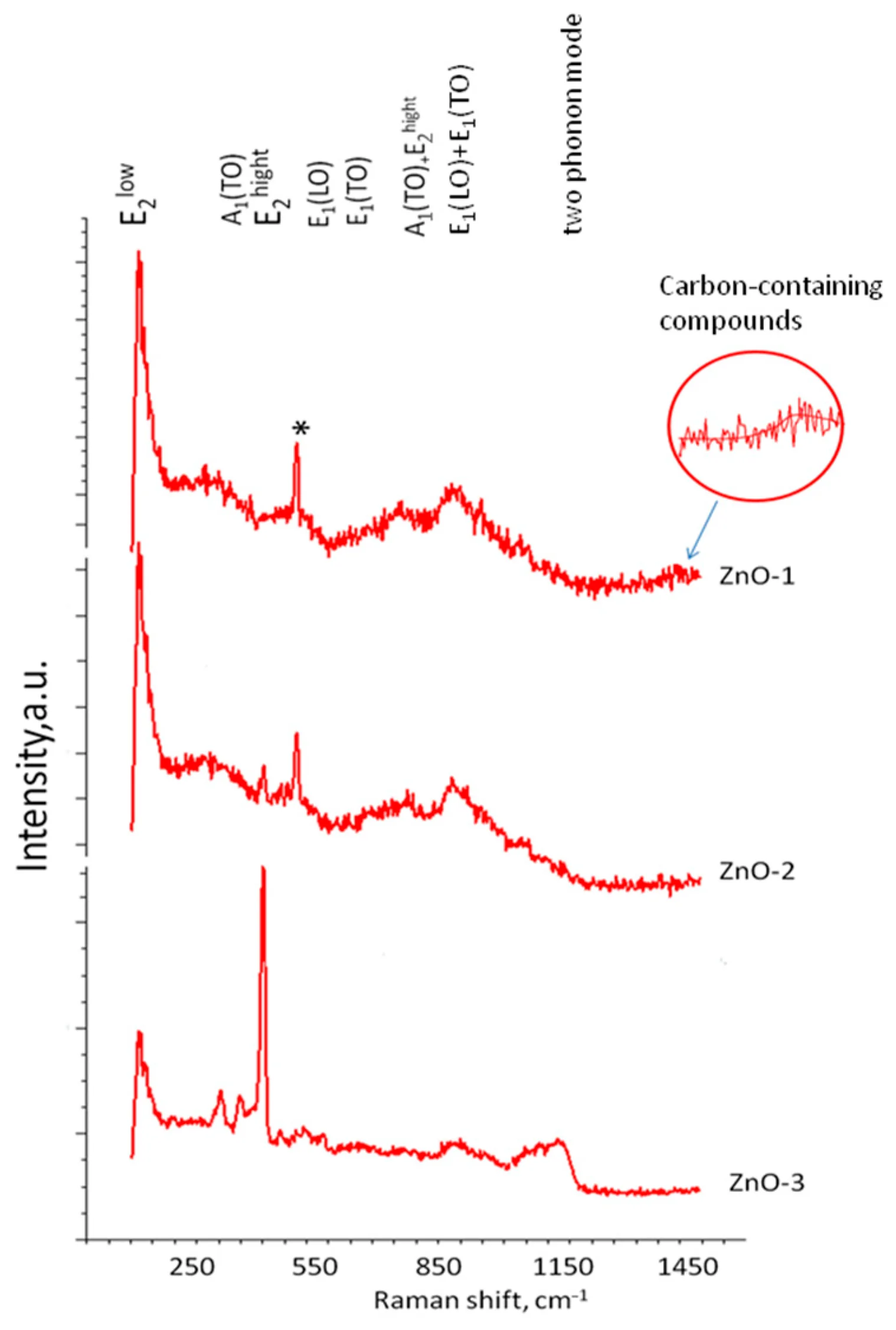
Raman spectra of the studied zinc oxide powders: 1—ZnO-1; 2—ZnO-2; 3—ZnO-3. Designation: *—silicon substrate peak.

ATR-FTIR (Figure 11) and Raman spectroscopy of the near-surface region (Figure 12) were performed for all three ZnO samples. In the spectrum of the ZnO-1 sample, in addition to a broad absorption band in the region of 3200–3600 cm^−1^, attributed to hydroxyl groups and adsorbed water, additional absorption bands are observed at ~1500, ~1400, ~835, and ~708 cm^−1^. This set of absorption bands is characteristic of zinc carbonate species on the particle surface. The bands in the region of 1500 cm^−1^ are attributed to asymmetric stretching vibrations of carbonate ions ν(CO_3_^2−^); the band at 1400 (1380–1420) cm^−1^ is attributed to the splitting of ν(CO_3_^2−^) vibrations (or the activation of ν(CO_3_^2−^) vibrations) due to the coordination of carbonate groups with Zn^2+^ ions. The bands around 835 and 708 cm^−1^ correspond, respectively, to out-of-plane δ_op_ (CO_3_^2−^) and in-plane d-bending δ_pl_ (CO_3_^2−^) vibrations of carbonate ions.

Raman features in ZnO powder are ascribed to Raman-active modes of the ZnO wurtzite crystal [46]. The zone center optical phonons can be classified according to the following irreducible representation: Γ_opt_ = *A*_1_ + *E*_1_ + 2*E*_2_ + 2*B*_1_, where *B*_1_ modes are silent, *A*_1_ and *E*_1_ are polar modes, both Raman and infrared active, while *E*_2_ modes (*E*_2_ low and *E*_2_ high) are non-polar and Raman active only. The characteristic E_2_^low^ and E_2_^high^ modes are clearly observed for the ZnO-2 and ZnO-3 powders (Figure 12). For the ZnO-1 powder, only the low-frequency E_2_^low^ mode is observed. The E_2_^low^ mode is influenced by Zn^2+^ interstitial defects, and the E_2_^high^ mode by O_2_ vacancies [46]. Thus, the Raman spectroscopy confirms the low quality of the ZnO-1 sample. In addition, in the 2450 cm^−1^ region, a low-intensity band associated with carbon-containing compounds [47] is observed.

The results of ATR-FTIR surface analysis and Raman spectroscopy confirm the presence of zinc carbonate and hydrocarbonate compounds on the powder surface.

In the micrograph of the ZnO-1 powder (Figure 1a), the particles exhibit a pronounced irregular shape, which indicates a low level of structural order. The primary particles are merged into large submicron agglomerates with loose packing and a high number of inter-particle voids, which additionally decreases the accessible surface and reduces the number of active sites.

The ZnO-2 powder, in contrast, is characterized by a relatively well-ordered crystal structure. In the micrograph (Figure 1b), plate-like and prismatic crystals with clearly defined faces and edges can be observed.

**Figure 13:**
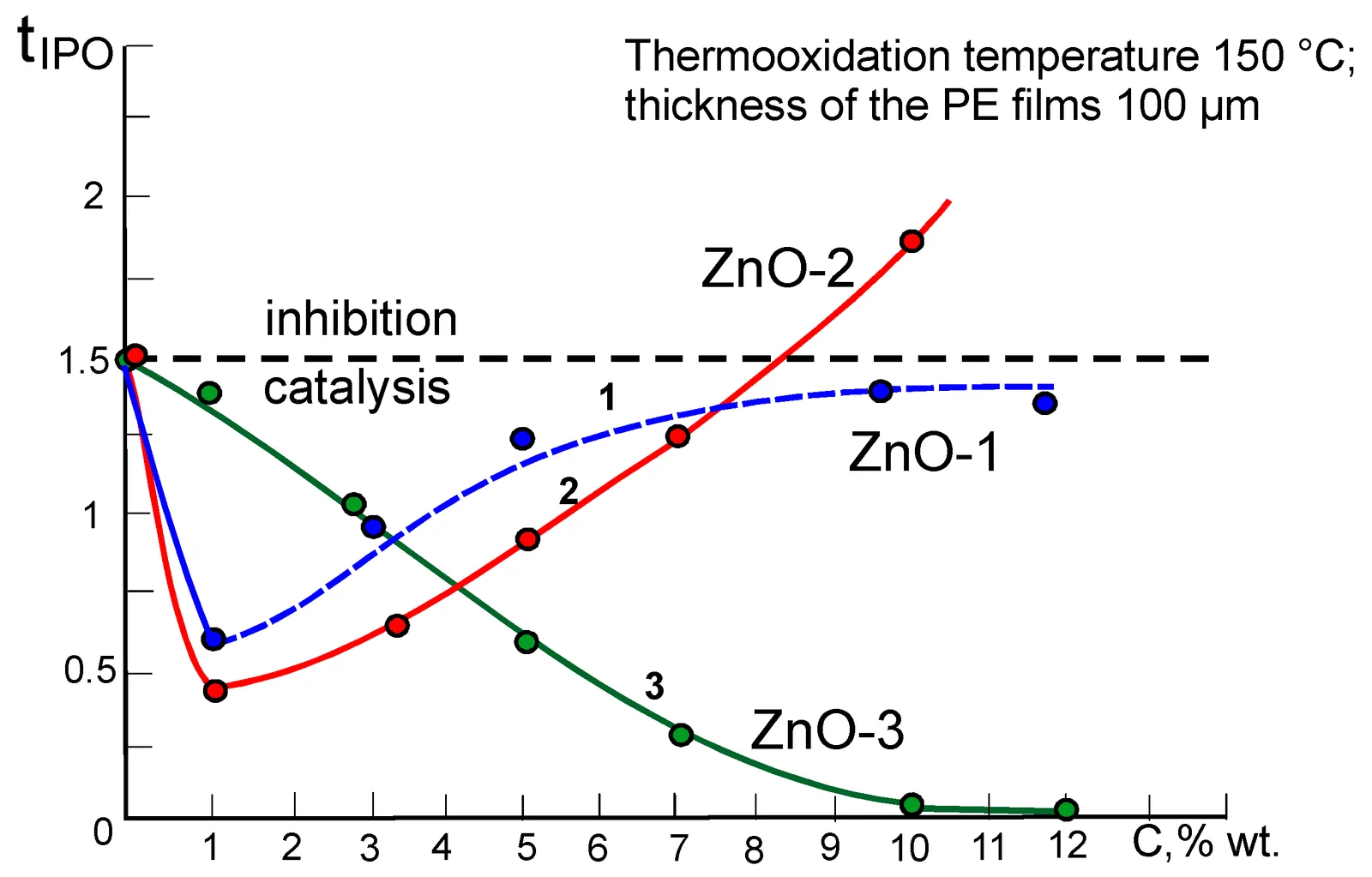
Dependence of the duration of OIP in PE films on ZnO concentration.

At high filler loadings, the tortuosity of oxygen diffusion pathways in the polymer matrix increases, hindering oxygen transport to reactive sites. Restricted oxygen diffusion reduces its participation in chain-propagation reactions, thereby increasing the OIP. Furthermore, higher filler concentrations increase the likelihood of ZnO particle agglomeration, leading to a higher local concentration of radical species at the ZnO/polymer interface and an increased probability of their bimolecular recombination. Therefore, the catalytic effect of the three ZnO powders should be evaluated by analyzing OIP as a function of powder concentration (Figure 13).

Notably, ZnO-1 particles at a concentration of 10 wt.% did not exhibit an inhibitory effect on the thermal oxidation of PE films (Figure 13, curve 1), unlike the larger ZnO-2 particles. This absence of inhibition is likely attributable to the combined effects of the small particle size and the presence of surface impurities. The high specific surface area of the nanoparticles provides a high density of accessible surface-active sites. However, carbonate and hydroxycarbonate species present on the ZnO-1 surface may partially block these sites. Consequently, no inhibition of PE oxidation is observed, even at high filler loadings.

The observed behavior can be attributed to the particle morphology. ZnO-3 powder, despite its larger particle size (10–15 μm), consists of randomly oriented needle- and plate-like crystals. Such a morphology reduces the likelihood of dense particle packing and the formation of stable agglomerates, ensuring the accessibility of the filler surface for interaction with oxygen and polyethylene macromolecules. In addition, the highly anisotropic particle morphology promotes the formation of less tortuous diffusion paths for oxygen (the reduced tortuosity). As a result, even at a high ZnO-3 content, sufficient oxygen supply to the ZnO/PE interfacial region and accessibility of catalytically active sites on the filler surface are maintained. This allows ZnO-3 to retain its catalytic activity and the accelerated thermal oxidation of PE films over a wide range of filler concentrations (Figure 13).

The experiment has shown that the catalytic activity of ZnO powders is determined not only by their dispersity and concentration, but also by a combination of additional factors, including particle morphology and impurity content.

### 2.4. Influence of ZnO Powder Morphology and Properties on the Methylene Blue Photocatalytic Degradation Processes

**Figure 14:**
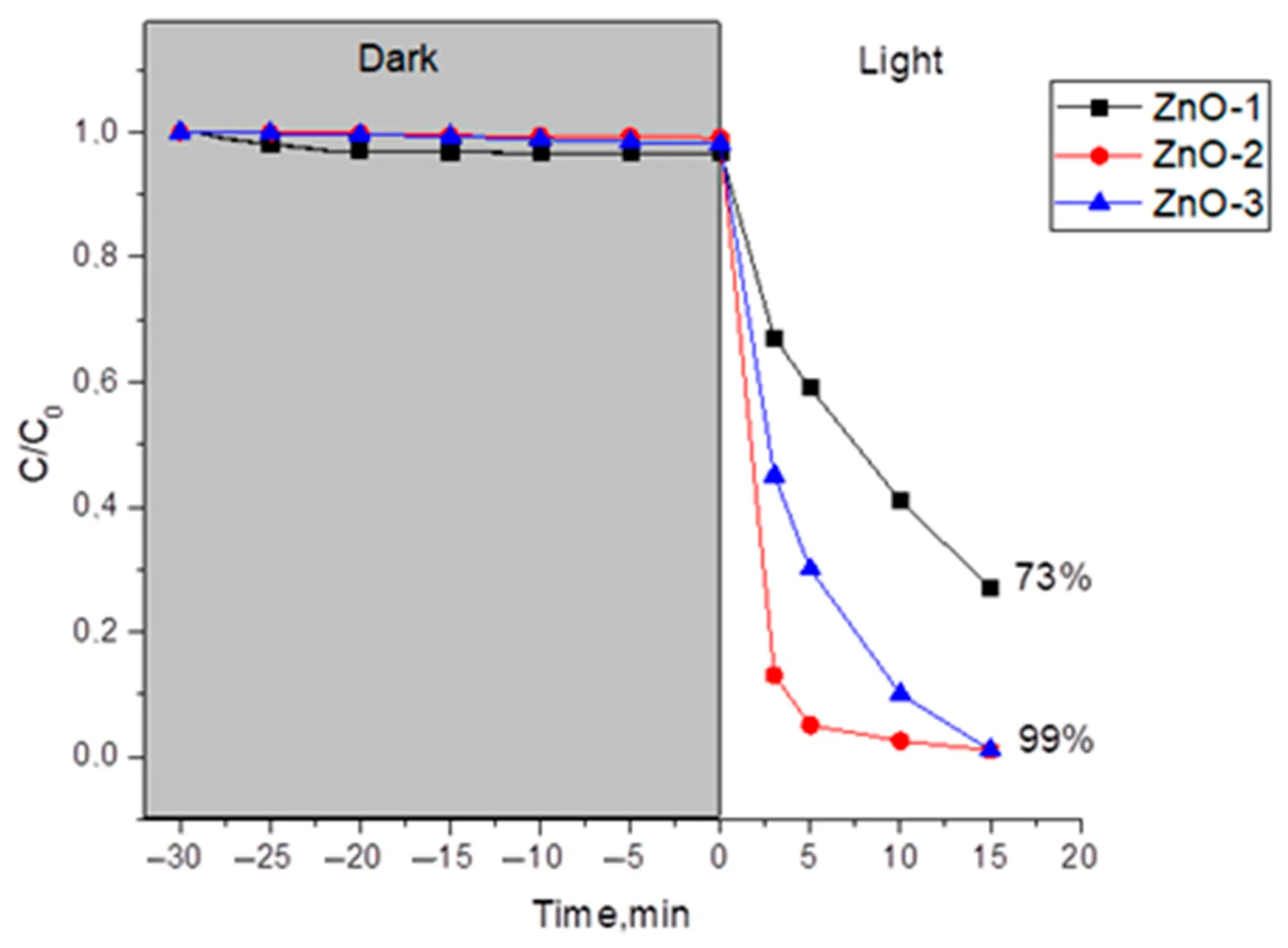
Photocatalytic degradation of methylene blue (MB, 1 mg/L) under light sources (Xe lamp, 150 W) using ZnO samples.

The dependences of C/C_0_ on the irradiation time in the presence of ZnO samples as photocatalysts are shown in Figure 14. Here, C_0_ and C are the MB concentrations in the solution before and after irradiation, respectively. At the initial step, the dark sorption of MB on ZnO powder particles was assessed. It did not exceed 4% and was highest for ZnO-1. The MB photodegradation rate constant k was calculated from the slope of the linearized kinetic curves ln(C/C_0_) versus t using the Langmuir–Hinshelwood model for low concentrations.

The maximum MB photodegradation rate was obtained for the ZnO-2 sample (k = 0.729 min^−1^). A somewhat slower process, especially at the initial stage, was observed for MB photodegradation in the presence of the ZnO-3 sample (k = 0.256 min^−1^). At the same time, the final efficiency of the ZnO-2 and ZnO-3 samples after 15 min was identical and reached 99%. The ZnO-1 sample demonstrated the lowest efficiency (k = 0.146 min^−1^). This process can be summarized by the following equations:

ZnO + hν → e^−^ + h^+^ (10)

e^−^ + O_2_ → ^•^O_2_^−^ (11)

h^+^ + H_2_O → ^•^OH + H^+^ (12)

^•^O_2_^−^ + H^+^ → HO_2_^•^ (13)

2HO_2_^•^ → H_2_O_2_ + O_2_ (14)

H_2_O_2_ + e^−^ → OH^−^ + ^•^OH (15)

h^+^ + OH^−^ → ^•^OH (16)

The photocatalytic activity of a catalyst during the degradation of organic colorants in solutions depends on a variety of factors, including the total surface area of the catalyst, the rate of charge carrier generation and their mobility, wettability, defect structure, and impurity content. In our case, all samples exhibited high wettability, including the ZnO-3 samples, despite their complex spatial morphology.

The binding of water molecules to the surface of ZnO microstructures occurs via ion–dipole interactions, with the dominant role played by polar and semi-polar crystal faces. In the ZnO-3 samples, the fraction of polar and semi-polar faces is high, and, taking into account the morphological irregularity of individual microstructures (i.e., the “lotus effect” is suppressed), a state of high wettability is achieved.

In catalysis, an important role is played by the catalyst–solution interface, and the presence of an extended interface should determine the catalytic activity. In our case, ZnO-1 nanoparticles, despite having a surface area many times larger, were characterized by the lowest rate constant, even compared to ZnO-3 microparticles. Obviously, the most significant factors are the presence of deep charge carrier traps, high defect density and the presence of impurities.

The ZnO-2 sample acts as a highly efficient photocatalyst: within the first three minutes, almost 90% of MB is degraded, and the subsequent decrease in the process rate is related to the diffusion-controlled supply of the contaminant.

During the photocatalysis, the ZnO-3 sample behaves somewhat differently from the ZnO-2 sample: a relatively rapid initial stage, caused by a high concentration of accessible active centers, is followed by a subsequent deceleration. The deceleration may be related to several factors, including the blocking of the active centers, their partial passivation, and the limitation of the diffusion-controlled supply.

**Figure 15:**
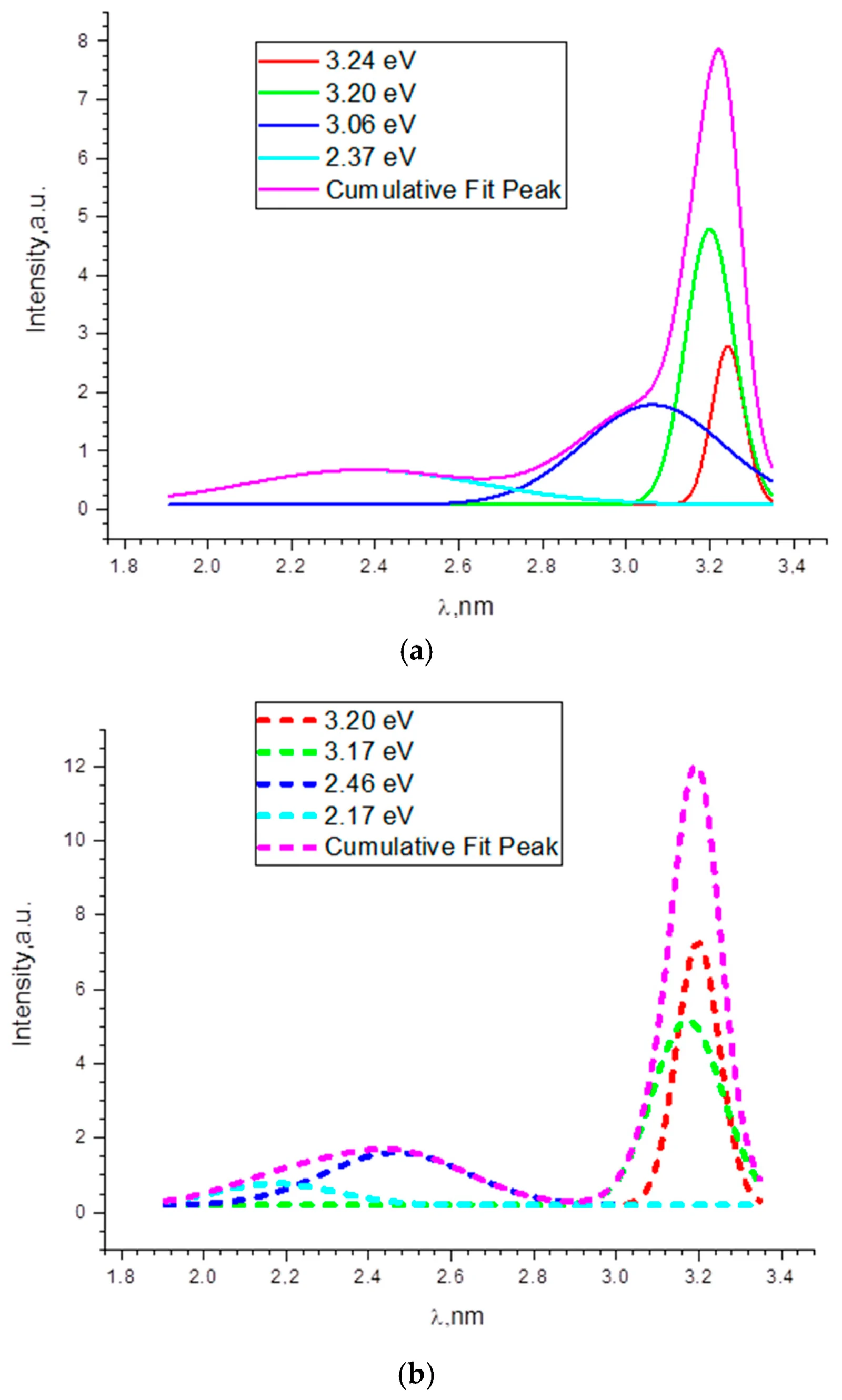
PL spectra of ZnO samples (excitation at λ = 315 nm): ZnO-2 (**a**); ZnO-3 (**b**).

Since intrinsic defects play a decisive role in the catalytic activity of ZnO, the PL spectra of ZnO-2 and ZnO-3 samples were analyzed (Figure 15). The correct presentation and interpretation of experimental spectroscopic data are important for understanding luminescence, and several common pitfalls have been discussed in [48]. To avoid erroneous interpretation, the PL spectra were presented on an energy scale, and we discuss only those emission bands whose assignments are widely accepted in the literature. In particular, the bands at 3.17 eV, 3.20 eV, and 3.24 eV are identified in ZnO-2 and ZnO-3 samples. These bands are most pronounced at low temperatures [49] and are attributed to free exciton emissions and phonon replicas. The band at 3.06 eV in ZnO is attributed to zinc vacancies V_Zn_ [50,51]. The bands in the region of 2.17–2.46 eV are commonly attributed to neutral and charged oxygen vacancies [52,53,54,55]. In the synthesized ZnO powders, V_o_ ^0^ and Zn_i_^0^ are stable, which are responsible for the n-type conductivity of ZnO at room temperature. Charged oxygen vacancies V_o_^++^ and V_o_^+^, which are formed during synthesis due to oxygen displacement into interstitial sites and its migration to the surface, are positively charged but are unstable in the bulk since ZnO is an n-type semiconductor. However, they can exist on the surface, being compensated by adsorbed gases. In solution, they can be stabilized by interactions with solvent molecules. Thus, oxygen vacancies are electron traps, while prolonging the lifetime of holes by trapping electrons. Zinc vacancies, on the contrary, are hole traps [56,57]. Photogenerated holes possess a much higher oxidation potential, so they can directly oxidize adsorbed molecules and promote the formation of hydroxyl radicals (^•^OH). Photogenerated electrons play a complementary role: they reduce O_2_ to superoxide radicals and participate in the formation of H_2_O_2_. Previous studies [57] have shown that surface oxygen defects are a key factor in the photocatalytic activity of ZnO. The low photocatalytic activity of ZnO-1 can be explained by the high density of grain boundaries, extended structural defects, and the presence of impurity-related defect centers that act as traps for both holes and electrons. The high photocatalytic activity of ZnO-2 and ZnO-3 samples is associated with the low content of impurities and extended crystal defects. Furthermore, point defects V_O_ and V_Zn_ increase the lifetime of charge carriers in ZnO-2, thereby enhancing its photocatalytic activity. All the results obtained in the work are summarized in Table 2.

Overall, the relationship between the photoluminescent and photocatalytic properties of the ZnO samples can be inferred from the data presented in Table 2: the photocatalytic rate constant correlates with the intensity ratio of the UV and GL band maxima. In addition, the lowest photocatalytic activity is exhibited by the ZnO-1 nanopowder, which shows suppressed luminescence due to the low crystalline quality and contamination by carbon-based impurities during synthesis. This is consistent with the XRD data for the ZnO-1 sample. However, the XRD data for the ZnO-2 and ZnO-3 samples demonstrate similar crystal structure parameters and therefore do not explain the pronounced difference in their photocatalytic activity.

The presence of a high-intensity UV PL band in the ZnO-2 sample indicates a high rate of charge carrier generation and recombination. In photocatalysis, in contrast, it is necessary to suppress the charge carrier recombination rate while maintaining a high generation rate, since the diffusion of charge carriers to the surface is critically important. In the ZnO-2 sample, the presence of charge carrier traps in the near-surface layers promotes the separation of electrons and holes diffusing to the surface.

In the PL spectra of ZnO-3, long-lived stages are absent; however, in contrast to ZnO-2, a defect-related band with an emission time of approximately 1 μs is present. In zinc oxide, such defect bands are associated with oxygen vacancies, interstitial zinc, interstitial oxygen, and their complexes, which act as deep charge carrier traps. Their presence slows both recombination processes and the diffusion of charge carriers to the surface. As a result, the I_UVL_/I_GL_ ratio in the ZnO-3 sample is reduced compared to that of the ZnO-2 sample. At the same time, on the surface of ZnO-3 samples, due to surface reconstruction after chemisorptions of MB molecules, recombination processes are accelerated. In addition, it should be borne in mind that the molecules of methylene blue are quite large: settling on the surface of zinc oxide, they tightly cover its surface and block it. Another factor contributing to the lower photocatalytic rate of the ZnO-3 sample is its relatively low specific surface area.

## 3. Materials and Methods

Three types of ZnO samples were used: ZnO-1—commercial nanoparticles; ZnO-2—commercial submicron particles; and ZnO-3—microparticles synthesized using plasma technologies. The ZnO microstructures were synthesized by an atmospheric-pressure microwave plasma method [3].

Microwave plasma synthesis of ZnO was carried out using a 2.45 GHz waveguide plasmatron operating at atmospheric pressure. The system comprised a magnetron source (up to 3 kW CW power), a WR-340 rectangular waveguide with a water-cooled load, and a quartz discharge tube (30 mm ID) mounted perpendicularly to the waveguide. High-purity nitrogen (99.998%) was supplied at ~10 L·min^−1^, carrying zinc powder (30–40 µm) through the plasma region.

Microscopic studies were conducted using a JCM-6000 (JEOL, Tokyo, Japan) scanning electron microscope (SEM) equipped with an energy-dispersive X-ray (EDX) detector. To ensure reliable determination of the carbon content by EDX, the powders were deposited directly onto a clean silicon substrate, thereby minimizing interference from carbon-containing mounting materials.

X-ray diffraction (XRD) patterns were obtained on an Empyrean diffractometer (PANalytical, Almelo, The Netherlands) in Bragg–Brentano geometry using Cu Kα radiation (λ = 1.54 Å). The specific surface area was determined by the multipoint BET method using a Sorbi^®^-MS instrument (Meta, Novosibirsk, Russia). Infrared spectra were recorded using ATR-FTIR spectroscopy on a Bruker VERTEX 70 FTIR spectrometer (Bruker Optics, Ettlingen, Germany). Powder samples were placed directly onto the ZnSe ATR crystal, and intimate contact between the sample and the crystal was ensured using a clamping device. Raman spectra were recorded using an Ntegra Spectra (NT-MDT, Zelenograd, Russia) with a 532 nm excitation laser, laser power of 5 mW, and a laser spot diameter of approximately 50 μm.

The photoluminescence (PL) spectra were recorded using a CM 2203 spectrofluorometer (SOL-Instruments, Minsk, Belarus) and a Fluorate-02-Panorama photoluminescence spectrometer. The analyzer light source was a high-pressure xenon lamp, operating in a pulsed regime, providing short excitation (1 μs) pulses at a repetition frequency of 25 Hz. The working spectral range of the analyzer was 210–280 nm. The instruments were equipped with two monochromators, one for the excitation and one for photoluminescence registration. A portion of light reflected from the beam-splitting plate was directed to the radiation receiver of an additional reference channel, which was used to correct the luminescence signal. To improve the reliability of the results, in addition to correction using a reference channel, signal averaging over the specified number of excitation pulses was applied.

The shape of the recorded luminescence pulse depends on the properties of the luminescent components and can either reproduce the shape of the excitation pulse (fluorescence) or be detected with a delay relative to the excitation pulse (phosphorescence).

For the measurements, a time interval was selected during which the luminescence signal intensity was accumulated. The detection strobe (up to 1000 μs) could be set with a specific delay relative to the leading edge of the synchronization pulse that triggered the lamp (up to 6000 μs).

The measurements were performed in a standard mode for registering luminescence with durations shorter than 1 μs (strobe duration of 4.95 μs and strobe delay of 0.95 μs), as well as with various strobe durations and delays for registering luminescence in the micro- and millisecond time ranges.

The sample was placed in the cuvette compartment at a 45-degree angle, i.e., in reflection mode, without absorption correction. All measurements were carried out at room temperature.

Samples for luminescence measurements were prepared in the form of powders lightly pressed onto a diaphragm to a layer thickness of ~1 mm. Since excitation was performed in the band-to-band transition region, where the transparency of the samples is limited, light scattering and absorption in the near-surface layer play a significant role in signal formation. The excitation and luminescence depths near the absorption edge (≤1 μm) increase with increasing wavelength, which leads to a reduction in the self-absorption effect.

The catalytic (or inhibiting) effect of ZnO powder particles on the thermo-oxidative degradation process was evaluated by changes in the oxidation induction period (OIP) of polyethylene (PE) films containing 1–12 wt.% ZnO at 150 °C.

A powdered, non-stabilized low-pressure PE (GOST 16338) with a weight-average molecular weight Mw = (0.5–0.7) × 10^5^ was used in the experiments.

PE and ZnO powders were first prepared, after which analytical-grade acetone was added. The resulting suspension was mixed using a magnetic stirrer (2–3 min), followed by ultrasonic dispersion (8 min). After complete evaporation of the acetone, PE films with a thickness of 100 μm were prepared by thermal pressing. The pressing parameters were as follows: temperature of 150 ± 5 °C, pressure of 80–100 kg/cm^2^, and a pressing time of up to 30 s.

PE films were fused onto IR-transparent KBr crystals. The samples were then placed in a thermostatic oven and subjected to thermo-oxidative treatment at 150 °C. At 0.5 h intervals, the PE films were removed from the oven, cooled to room temperature, and analyzed by IR spectroscopy. The IR spectra were recorded using a Vertex 70 Fourier-transform IR spectrometer (Bruker, Ettlingen, Germany) in transmission mode over the spectral range of 4000–500 cm^−1^ with an optical resolution of 4 cm^−1^.

The degree of oxidation of PE films was evaluated using the optical density of the 1720 cm^−1^ band, which is attributed to the valent oscillations of carbonyl groups. The absorption band at 1460 cm^−1^, associated with the bending oscillations of methylene groups, was used as an internal reference.

The optical density of the 1720 cm^−1^ band was calculated as a ratio of the integrated peak areas within the ranges of 1840–1670 cm^−1^ (maximum at 1720 cm^−1^) and 1505–1390 cm^−1^ (maximum at 1465 cm^−1^). All spectra were preliminary baseline corrected to ensure accurate integration and comparability of the results. The relative error of the peak area determination, estimated from three repeated scans, did not exceed 3%.

The OIP of the PE films was determined graphically from the cumulative kinetic curves of carbonyl groups. The values of the optical density of the 1720 cm^−1^ band reported in this study are presented as arithmetic mean values. Each value was obtained from 3 to 4 parallel measurements performed on the same experimental film sample.

For the photocatalytic activity measurements, ZnO samples (10 mg) were dispersed in 25 mL of methylene blue (MB) solution with a concentration of 1 mg/L. The suspension was then stirred in the dark for 30 min and subsequently irradiated under continuous stirring. A xenon lamp OLKs-150M (Optotekhnika LLC, Saint Petersburg, Russia) was used as a light source with an irradiation power of 150 W and an intensity of ~50 mW/cm^−2^. The MB concentration was determined by a spectrophotometric method using the maximum optical density at 664.7 nm, measured with a PB 2201 spectrophotometer (SOL-Instruments, Belarus).

## 4. Conclusions

In this work, a comprehensive analysis of the relationships among the photoluminescent, thermo-oxidative, and photocatalytic properties (under simulated solar irradiation) of ZnO powders was carried out, and their correlations with X-ray diffraction and microscopic characterization data were investigated. ZnO powders of various sizes and morphologies were used: pseudospherical nanoparticles (30–50 nm), submicron (100–500 nm) faceted crystals, and microstructures (up to 20 μm) in the form of rod-like crystals and plates. The present results demonstrate that the catalytic activity of ZnO powders is determined not only by their dispersion state and concentration, but also by a combination of additional factors: particle morphology, crystalline quality, and the presence of impurities. Despite the different mechanisms of photocatalytic and thermo-oxidative processes, their efficiencies are largely governed by the surface state of ZnO particles and the concentration of surface-active sites. Photoluminescence spectroscopy is a sensitive method for evaluating the surface state of ZnO, since the overall luminescence intensity, the intensity ratio, and the spectral position of individual emission bands reflect the structural quality and the concentration of point defects in the crystal lattice. The presence of an intense ultraviolet emission band and a high overall luminescence intensity indicates a high rate of generation of electron–hole pairs and a low concentration of deep carrier trapping centers. Point defects, which are responsible for the green luminescence band, not only prolong the lifetime of photogenerated charge carriers by suppressing their recombination but also act as active sites for oxygen adsorption, catalytic hydroperoxide decomposition, and high-temperature dehydrogenation reactions. Therefore, photoluminescence analysis represents a simple predictive tool for indirectly assessing the potential catalytic activity of ZnO powders in both photooxidation and thermo-oxidative processes.

It can be concluded that the structural and morphological characteristics of zinc oxide powder (particle size, degree of crystallinity, defect concentration, specific surface area, presence of impurities) determine its thermo-oxidative and photocatalytic performance. To objectively evaluate these parameters, a set of complementary methods was used. A comparison of data obtained using different methods shows good agreement and allows the following conclusion to be drawn: it is precisely the optimization of the ZnO structure, and not any individual parameter, that can ensure high catalytic activity. The observed relationships can serve as a basis for the targeted synthesis of effective zinc oxide-based catalysts.

## Figures and Tables

**Table 1:** Lattice parameters (a, c), lattice volume (V) and its deviation from the standard (∆V), calculated values of crystallite size (D) and microstrain (ε) of ZnO nanoparticles of different types.

Sample Types	a, Å	c, Å	c/a	V, Å^3^	∆V, %	D, nm	ε·10^3^
1	3.248 ± 0.001	5.207 ± 0.001	1.603 ± 0.001	47.58 ± 0.03	−0.01	35.8	0.25
2	3.245 ± 0.001	5.208 ± 0.001	1.605 ± 0.001	47.49 ± 0.03	−0.28	68.4	0.02
3	3.245 ± 0.001	5.206 ± 0.001	1.604 ± 0.001	47.48 ± 0.03	−0.30	69.3	0.02

**Table 2:** Photocatalytic and photoluminescent properties of samples.

Sample	Specific Surface Area, m^2^/g	OIP in PE Films, 1 wt.% ZnO, Hours	OIP in PE Films, 10 wt.% ZnO, Hours	The Nature of the Catalytic Effect Depending on the Concentration	Rate Constant, min^−1^	I_UV_/I_GL_
Type 1	32	0.6 (active catalysis)	1.4 (very weak catalysis)	At 1%, acceleration is maximum. As concentration increases, activity decreases, but inhibition is not observed (even at high concentrations).	0.146	-
Type 2	3.8	0.4 (active catalysis)	1.8 (inhibition)	At 1%, acceleration is maximum. As concentration increases, activity decreases, and at ≥10 wt.%, oxidation is inhibited.	0.729	11.6
Type 3	2.6	1.3 (weak catalysis)	0 (active catalysis)	At low concentrations (1%), oxidation is weakly affected. Maximum catalytic activity occurs at high concentrations (10–12%).	0.256	6.88

## Data Availability

The original contributions presented in this study are included in the article. Further inquiries can be directed to the corresponding author.
